# A Chiral Bipyrimidine-Bridged Dy_2_ SMM: A Comparative Experimental and Theoretical Study of the Correlation Between the Distortion of the DyO6N2 Coordination Sphere and the Anisotropy Barrier

**DOI:** 10.3389/fchem.2018.00537

**Published:** 2018-11-08

**Authors:** Ismael F. Díaz-Ortega, Juan Manuel Herrera, Álvaro Reyes Carmona, José Ramón Galán-Mascarós, Sourav Dey, Hiroyuki Nojiri, Gopalan Rajaraman, Enrique Colacio

**Affiliations:** ^1^Departamento de Química Inorgánica, Facultad de Ciencias, Universidad de Granada, Granada, Spain; ^2^Institute of Chemical Research of Catalonia, The Barcelona Institute of Science and Technology, Tarragona, Spain; ^3^Institució Catalana de Recerca i Estudis Avançats (ICREA), Barcelona, Spain; ^4^Department of Chemistry, Indian Institute of Technology Bombay, Mumbai, India; ^5^Institute for Materials Research, Tohoku University, Sendai, Japan

**Keywords:** chiral, SMMs, Dy_2_, bipyrimidine-bridged, diketonates, *ab initio* calculations, magnetic properties

## Abstract

Chiral bipyrimidine-bridged dinuclear Ln^III^ complexes of general formula [(μ-bipym){((+)-tfacam)_3_Ln}_2_] and [(μ-bipym){((-)-tfacam)_3_Ln}_2_], have been prepared from the assembly of Ln(AcO)_3_·nH_2_O (Ln^III^ = Dy, Gd), (+)/(−)-3-(trifluoroacetyl)camphor enantiopure ligands ((+)/(-)-Htfacam) and bipyrimidine (bipym). The structure and chirality of these complexes have been supported by single-crystal X-Ray diffraction and circular dichroism. The study of the magnetic properties of the Gd^III^ complexes revealed a very weak antiferromagnetic interaction between the Gd^III^ ions through the bipyrimidine bridging ligand. *Ab initio* CASSCF calculations indicated that the ground Kramers doublet (KD) of both Dy^III^ centers is almost purely axial with the anisotropy axis located close to the two tfacam^−^ligands at opposite sides of each Dy^III^atom, which create an axial crystal field. In keeping with this, *ac* dynamic measurements indicated slow relaxation of the magnetization at zero field with *U*_eff_ = 55.1 K, a pre-exponential factor of τ_o_ = 2.17·10^−6^ s and τ_QTM_ = 8 μs. When an optimal dc field of 0.1 T is applied, QTM is quenched and *U*_eff_ increases to 75.9 K with τ_o_ = 6.16 × 10^−7^ s. The DyN_2_O_8_ coordination spheres and SMM properties of [(μ-bipym){((+)-tfacam)_3_Ln}_2_] and their achiral [(Dy(β-diketonate)_3_)_2_(μ-bpym)]analogous have been compared and a magneto-structural correlation has been established, which has been supported by theoretical calculations. In contrast to the Gd^III^ compounds, the magnetic exchange interaction between the Dy^III^ ions has been calculated to be very weak and, generally, ferromagnetic in nature. Relaxation mechanisms for [(μ-bipym){((+)-tfacam)_3_Ln}_2_] and previously reported analogous have been proposed from *ab initio* calculations. As the magnetic exchange interaction found to be very weak, the observed magnetization blockade in these systems are primarily dictated by the single ion anisotropy of Dy^III^ ions.

## Introduction

During the last few decades, molecular materials in which coexist two or more physical properties (multifunctional molecular materials) have attracted much attention mainly due to their promising potential applications (Coronado et al., 2003a,b, 2008; Gómez-Romero and Sánchez, 2004; Fahmi et al., 2009; Rocha et al., 2011; Sanchez et al., 2011; Ouahab, 2012). It is well-known that a molecule without any improper axis of symmetry (S_n_) is chiral, that is to say is not superimposable with its mirror image (enantiomer). Interestingly, each enantiomer can interact in a different manner with the environment. Thus, for instance, enantiomers are optically active and rotate the plane of polarized light the same angle but in opposite directions. In multifunctional materials based on coordination compounds, chirality can be spawn by using enantiomerically pure ligands (Amouri and Gruselle, 2008; Pinkowicz et al., 2015). The inherent chirality of the ligand may introduce additional functions to coordination compounds with interesting magnetic properties such as magnetochiral dichroism (MChD) effect (Rikken and Raupach, 1997, 2000; Train et al., 2008, 2011), second harmonic generation (SHG) (Bogani et al., 2006; Train et al., 2009), and ferroelectric properties (Wang et al., 2012; Li et al., 2017). It has been recently shown that mononuclear tris(β-diketonate) Dy^III^ complexes containing a N,N-bidentate chelate aromatic ligand, such as 2,2′-bipyrimidine and 1,10-phenanthroline derivatives, as well as Dy_2_dinuclear complexes containing bis(didentate) bridging ligands connecting two tris(β-diketonate) Dy^III^ moieties, such as 2,2'-bipyrimidine and 2,2'-bipyrimidine-N-oxide, exhibit Single-Molecule Magnet (SMM) behavior at zero field with significant thermal energy barriers (*U*_eff_) (Chen et al., 2011, 2012; Wang et al., 2013; Tong et al., 2015; Sun et al., 2016; Yu et al., 2016; Cen et al., 2017; Díaz-Ortega et al., 2018). SMMs are nanomagnets that, in addition to the classical properties of a magnet, such as freezing of magnetization and magnetic hysteresis below the so called blocking temperature (T_B_), exhibit interesting quantum properties, such as quantum tunneling of the magnetization (QTM) and quantum phase interference (Aromí and Brechin, 2006; Gatteschi et al., 2006; Andruh et al., 2009; Bagai and Christou, 2009; Sessoli and Powell, 2009; Brechin, 2010; Guo et al., 2011; Rinehart and Long, 2011; Sorace et al., 2011; Clemente-Juan et al., 2012; Luzon and Sessoli, 2012; Wang and Gao, 2012; Habib and Murugesu, 2013; Woodruff et al., 2013; Zhang et al., 2013; Bartolomé et al., 2014; Layfield, 2014; Sharples and Collison, 2014; Craig and Murrie, 2015; Layfield and Murugesu, 2015; Liddle and van Slageren, 2015; Rosado Piquer and Sañudo, 2015; Tang and Zhang, 2015; Frost et al., 2016; Harriman and Murugesu, 2016). SMMs are of current interest not only due to the above indicated outstanding physical properties but also due to their envisaged applications in molecular spintronics (Bogani and Wernsdorfer, 2008; Dediu et al., 2009; Mannini et al., 2010; Vincent et al., 2012; Ganzhorn et al., 2013; Jenkins et al., 2013; Prezioso et al., 2013; Thiele et al., 2014; Lumetti et al., 2016; Cornia and Seneor, 2017), ultra-high density magnetic information storage (Rocha et al., 2005; Affronte, 2009), magneto-optics (Sessoli et al., 2015) and as qubits for quantum computing at molecular level (Leuenberger and Loss, 2001; Ardavan et al., 2007; Stamp and Gaita-Ariño, 2009; Martínez-Pérez et al., 2012; Ghirri et al., 2017). SMM behavior is bound to the existence of an energy barrier (*U*) that avoid magnetization reversal when the polarizing field is suppressed. In principle, in the absence of QTM and TA-QTM (thermal activated as quantum tunneling of the magnetization), which shortcut the relaxation barrier to an effective value (*U*_eff_), T_B_ increases with the height of the energy barrier. As the height of *U* primarily depends on the magnetic anisotropy, researchers have focused their attention on complexes containing lanthanide ions (and actinide) (Chen et al., 2016, 2017; Ding et al., 2016; Gregson et al., 2016; Gupta et al., 2016a; Liu et al., 2016; Goodwin et al., 2017; Guo et al., 2017), which exhibit large intrinsic magnetic anisotropy and large magnetic moments in the ground state. The use of lanthanide ions and, particularly the Dy^III^ ion (a Kramers ion for which QTM should be forbidden in the absence of magnetic field) has led to metal complexes with higher energy barriers and improved SMM properties. In polymetallic Ln complexes, Ln···Ln interactions increase quantum tunneling rates, leading to apparently low U_eff_ values. However, it has been shown that weak intramolecular Dy···Dy magnetic interactions in dinuclear Dy_2_ complexes do not eliminate the barrier for magnetization reversal when anisotropic axes are parallel (Moreno Pineda et al., 2014). Recently, it has been also shown that the incorporation of a Dy_2_ unit with an strong Dy-electron coupling inside a fullerene cage gives rise to a blocking temperature of 18 K. This fact demonstrate that not only the magnetic exchange between the Dy^III^ ions but also the ligand environment is crucial in dictating both the effective energy barrier and eventually the blocking temperature for this structural motif (Singh et al., 2015; Singh and Rajaraman, 2016; Liu F. et al., 2017).

The ligand 2,2'-bipyrimidine (bipym) has been shown to exhibit a great ability to bridge metal ions affording homopolynuclear d and f complexes, as well as heteropolynuclear d/d and d/f complexes. In these complexes, bipym is able to transmit moderate to strong magnetic exchange interactions between the metal ions in d/d complexes and very weak magnetic coupling in f/f systems (De Munno et al., 1996; Znovjyaka et al., 2009; Visinescu et al., 2010). Moreover, bipym is able to sensitize the luminescence of the lanthanide ions in the Vis and near IR regions (Zucchi, 2011).

In view of the above considerations, the assembly of chiral tris(β-diketonate) Dy^III^ frameworks (where the chirality is introduced by chiral β-diketonates) and N_4_-bis(bidentate) bridging ligands can be a good strategy to obtain chiral dinuclear Dy_2_ SMMs. Moreover, the magnetic interactions (exchange and dipolar) between the Dy^III^ ions transmitted by the bridging ligand can contribute to quench QTM and to observe the real energy barrier (*U*) for magnetization reversal. It is worth mentioning that the examples of coordination compounds where SMM and chirality coexist are scarce (Domingo et al., 2003; Singh et al., 2009; Inglis et al., 2011; Novitchi et al., 2011, 2012; Zhu et al., 2011, 2014; Li et al., 2012; Wang et al., 2014; Ou-Yang et al., 2016; Wada et al., 2016; Escuer et al., 2017; Fernandez-Garcia et al., 2017; Lippert et al., 2017; Liu M.-J. et al., 2017; Peng et al., 2017; Wen et al., 2018).

This paper reports the synthesis, crystal structure, detailed ac and dc magnetic studies and *ab initio* theoretical calculations of the dinuclear complexes [(μ-bipym){((+)-tfacam)_3_Dy}_2_] (d-**1**) and [(μ-bipym){((-)-tfacam)_3_Dy}_2_] (l-**1**), where (+)/(-)-Htfacam are (+)/(–)-3-(trifluoroacetyl)camphor enantiopure ligands and bipym is the bipyrimidine bridging ligand. It is worth mentioning that three achiral [(μ-bipym){(β-diketonate)_3_Dy}_2_]complexes have been previously prepared where β-diketonate = dibenzoylmethane(HDbzm) and 2,2,6,6-tetramethyl-3,5-heptanedioneand (Htmh) (Sun et al., 2016; Yu et al., 2016). The complex [(μ-bipym){(Dbzm)_3_Dy}_2_]·2CH_3_Cl (**2**) (Sun et al., 2016) displays two relaxation processes with *U*_eff_ = 201 and 67 K at zero field, while the analogous complex with different crystal solvent molecules [(μ-bipym){(Dbzm)_3_Dy}_2_]·MeCN (**3**) (Sun et al., 2016) and the complex [(μ-bipym){(tmh)_3_Dy}_2_](**4**) (Yu et al., 2016) exhibit only a relaxation process with *U*_eff_ = 267 and 97 K, respectively. In view of these results, the SMM behavior of **1** appears to be guaranteed. The aim of this study is threefold (i) to obtain a new example of chiral Dy_2_ SMM and to analyze if it presents ferroelectricity (ii) to compare its experimental and calculated relaxation mechanisms and their associated parameters with those previously obtained for analogous [(μ-bipym){(β-diketonate)_3_Dy}_2_] complexes and (iii) to draw useful conclusions for future development of the field (Graphical Abstract).

**Graphical Abstract:**
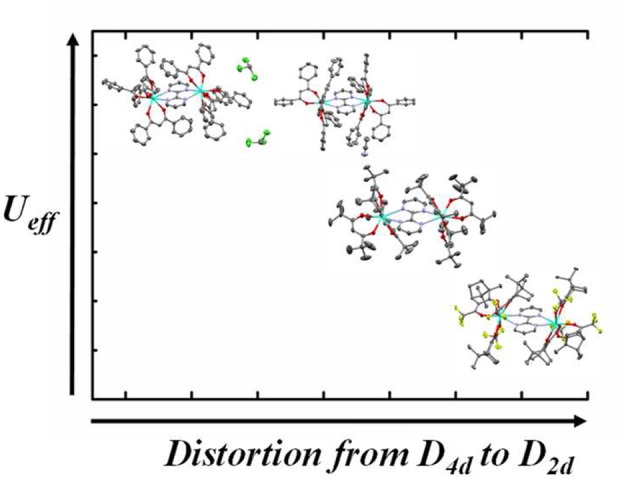
From theoretical and experimental results a correlation between the distortion of the DyO_6_N_2_ coordination sphere and the height of the anisotropy barrier has been established for a series of bipyrimidine-bridged Dy_2_ SMMs.

## Experimental section

### General procedures

Bipyrimidine, (+)/(–)-3-(trifluoroacetyl) camphor, solvents, and Dy(OAc)_3_·nH_2_O were purchased from commercial sources and used as received.

### Preparation of complexes

**[(**μ**-bipym){((**+**)-tfacam)**_3_**Dy}**_2_**]** (d-**1**): 0.50 mmol of Dy(AcO)_3_·nH_2_O dissolved in methanol (10 mL) were added dropwise to a solution of 2, 2′-bypirimidine (0.025 mmol) in methanol (10 mL). The solution was stirred for 10 min. and then added dropwise to a solution of d-Htfacam (0.150 mmol) in methanol (5 mL). The resulting solution was allowed to stand at room temperature. Partial evaporation of the solvent afforded a good crop of d-**1** as pale-yellow single crystals which were filtered, washed with a minimum amount of ethanol and air dried. Yield *ca*. 67%. Anal. calc. for [d-1] C_80_H_90_N_4_Dy_2_F_18_O_12_: C, 48.86; H, 4.61; N, 2.84. Found: C, 48.51; H, 5.41; N, 2.93.

**[(**μ**-bipym){((-)-tfacam)**_3_**Dy}**_2_**]** (l-**1**): Pale-yellow crystals of (l-**1**) were prepared by a method similar to that of d-**1**, except that (–)-3- (trifluoroacetyl)camphor (l-Htfacam) was employed instead of (+)-3-trifluoroacetyl)camphor(d-Htfacam).Yield *ca*. 60 %. Anal. calc. for C_80_H_90_N_4_Dy_2_F_18_O_12_: C, 48.86; H, 4.61; N, 2.84. Found: C, 48.59; H, 5.19; N, 2.88.

The Gd^III^complex**[(**μ**-bipym){((**+**)-tfacam)**_3_**Gd}**_2_**]** (d-**2**) was prepared by a similar method to that indicated above for **[(**μ**-bipym){((**+**)-tfacam)**_3_**Dy}**_2_**]** (d-**1**) but using a Gd(AcO)_3_·nH_2_O salt instead of the Dy(AcO)_3_·nH_2_O.Yield *ca*. 63 %. Anal. calc. for C_80_H_90_N_4_Gd_2_F_18_O_12_: C, 49.2; H, 4.63; N, 2.86. Found: C, 49.15; H, 5.01; N, 2.58.

**[(**μ**-bipym){((**+**)-tfacam)**_3_**Dy}**_2_**]** (d-**1**'). This diluted complex was prepared by following the same method as for **1** but using 0.024 mmol of Dy(AcO)_3_·4H_2_O and 0.476 mmol of Y(AcO)_3_·4H_2_O instead of 0.50 mmol of Dy(AcO)_3_·4H_2_O. The colorless crystal of d-**1**' were obtained with a yield of 60%. Anal. Calc. for [d-**1**'] C_80_H_90_N_4_Y_1.90_Dy_0.1_F_18_O_12_: C, 52.56; H, 4.93; N, 3.06. Found: C,.52.21; H, 5.04; N, 2.92. The Ir spectra of compounds d-**1**, l-**1**, d-**2**, and d-**1'** are virtually identical. Ir (cm^−1^): 2,964(m), 2,910 (w), 2,820(w), 1,624 (s), 150 (m), 1,541(m), 1,411(m), 1,267(m), 1,223(m), 1,187 (m), 1,128 (m), 1,079(m), 1,052(m), 920(m).

### X-ray crystallography

Suitable crystals of complexes d-**1**, l-**1**, and d-**2** were mounted on a Bruker D8 Venture (Mo Kα radiation, λ = 0.71073 Å, Photon 100 CMOS detector). Details of the crystals, data collection and refinement parameters are given as (Table S1). Once the data were processed (raw data integration, merging of equivalent reflections and empirical correction of the absorption), the structures were solved by either Patterson or Direct methods and refined by full-matrix least-squares on weighted F^2^ values using the SHELX suite of programs (Sheldrick, 2007) integrated in Olex2 (Dolomanov et al., 2009). Selected bond lengths and angles can also be found in (Tables S2–S4). Due to the poor quality of the data and crystallographic disorder affecting tfacam ligands the complete resolution of d-**2** was not possible. However, the unit cell [triclinic, *P*1, *a* = 100356 (6) Å, *b* = 12.9983 (8) Å, *c* = 17.4831 (10) Å, α = 101.7875 (19)°, β = 103.1306 (18)°, γ = 106.5972 (18)°, a partial refinement, and comparison between the experimental X-ray powder diffraction diagrams of d-**1** and d-**2** (Figure S8) confirm that both complexes are isostructural. To carry out the XRPD experiments, crystals of d-**1** and d-**2** were ground and deposited in the sample holder of a θ:θ Bruker AXS D8 vertical scan diffractometer. The generator was operated at 40 kV and 40 mA. The scans were performed with 4° < 2θ < 30° with *t* = 2 s and Δ2θ = 0.005°. CCDC-1834837-8 contains the supplementary crystallographic data for this article. These data are provided free of charge by the Cambridge Crystallographic Data Centre.

### Physical measurements

Elemental analyses were carried out at the “Centro de Instrumentación Científica” of the University of Granada on a Fisons-Carlo Erba analyser model EA 1108. FT-IR spectra were recorded with a Bruker Tensor 27 spectrometer using an ATR accessory. Direct (dc) and alternating (ac) current susceptibility measurements were performed with a Quantum Design SQUID MPMS XL-5 device. Ac experiments were performed using an oscillating *ac* field of 3.5 Oe and frequencies ranging from 1 to 1,500 Hz. Low-temperature magnetization measurements were performed by means of a conventional inductive probe in pulsed-magnetic fields. The temperature was reached as low as 0.4 K using a ^3^He cryostat (Nojiri et al., 2007). Polycrystalline specimens were mounted in a capillary tube made of polyimide. Samples of approximately 20 mg were not fixed within the sample tube and then they aligned along the magnetic field direction. Subsequently, a magnetic field was applied several times until orientation effect was saturated and the magnetization curves obtained in further shots were found to be identical. Solid-state CD spectra were performed on a JASCO J-810 spectropolarimeter at room temperature. Crystalline samples were ground to fine powders with potassium chloride and compressed into transparent disks. The concentration of the disks was 1.00 mg per 100 mg (sample/KCl) for CD spectra measurements. The P-E hysteresis loop was recorded with a home-made modified Sawyer-Tower circuit, using a reference capacitor (4.7 nF) and a digital Picoscope 1004 oscilloscope. The circuit was calibrated with monocrystalline BaTiO_3_.

### Computational methodology

MOLCAS 8.0 program package (Karlstrom et al., 2003; Veryazov et al., 2004; Duncan, 2009; Aquilante et al., 2010, 2016) has been used to perform post-Hartree-Fock*ab initio* calculations. Diamagnetic substitution method was followed to calculate the magnetic anisotropy on one Dy center while another Dy center was substituted by Lu. Basis set of VTZP quality was used for all the metals and atoms attached in the first coordination sphere of the metal and for rest of the atoms basis set of VDZ quality was used. All the basis set was taken from ANO-RCC (atomic natural orbital type with relativistic core corrections) library implemented in MOLCAS8.0 software (Aquilante et al., 2010). Using DKH Hamiltonian relativistic effects was treated in two steps. For the generation of basis sets scalar terms were included which have been used to determine spin-free wave functions and also energies through the use of the complete active space self-consistent field (CASSCF) method (Chibotaru and Ungur, 2012). Thus, spin-orbit free states were obtained by employing the RASSCF method whereas spin-orbit coupling has been taken into account using RASSI-SO method (Habib et al., 2013; Langley et al., 2014) which uses CASSCF wave functions as the basis sets and multiconfigurational wave functions as input states. The resulting wave functions and the energies of the molecular multiplets were used for the calculation of the magnetic properties and g tensors of the lowest state using a specially designed routine SINGLE_ANISO (Roos et al., 2008). As a consequence, the magnetic properties of a single magnetic ion are calculated by a fully *ab initio* approach in which the spin-orbit coupling is considered non-perturbatively. The active space consists all the 4f electrons in seven orbital CAS(9,7) which generates 21 sextet states. The consideration of only these states is sufficient to reproduce the experimental measurements as it has been seen from the previous studies (Upadhyay et al., 2014, 2017; Gupta et al., 2016c; Mukherjee et al., 2016; Vignesh et al., 2017a,b). In order to save disk space, Cholesky decomposition possessing a threshold of 0.2^*^10^−7^ has been incorporated for our calculations (Koch et al., 2003). Magnetic exchange interactions, exchange spectrum and all other magnetic properties of Dy_2_ dinuclear complex has been deduced using Lines model within the POLY_ANISO (Ungur and Chibotaru, 2007) routine which interfaced with SINGLE_ANISO based on the *ab initio* results of individual metal fragments (Feltham et al., 2011; Ungur and Chibotaru, 2011; Ungur et al., 2013).

To calculate the magnetic exchange between the Dy ions DFT calculations has been performed with hybrid B3LYP functional using Gaussian 09 programme (Schafer et al., 1992, 1994; Becke, 1993; Frisch et al., 2016). Since Dy(III) possess first order spin orbit coupling due to weak splitting of 4f orbitals, it cannot be described by single determinant. To get the remedy of these problem Dy atoms was replaced by Gd atoms since it does not contain first order orbital angular momentum and can be described as a single determinant. We have employed Cundari-Stevens (CS) relativistic effective core potential for Gd atom, TZV basis set for the atoms in the first coordination sphere around metal ion (Scuseria and Schaefer, 1989; Schafer et al., 1992, 1994; Cundari and Stevens, 1993). From the second coordination onwards a basis bet of SVP quality was used (Cundari and Stevens, 1993; Hänninen et al., 2018). Quadratic convergence method was followed to the most stable wave function. The energy of the high spin state was obtained from the single determinant approach while the energy of the BS state was obtained by the approach developed by Noodleman (Noodleman, 1981; Hänninen et al., 2018). Exchange is calculated from the energy difference of the BS and HS states. To find the exchange coupling constant between Dy ions resulting exchange was multiplied by a factor of 5/7.

## Results and discussion

### Syntheses and crystal structures

**Scheme 1:**
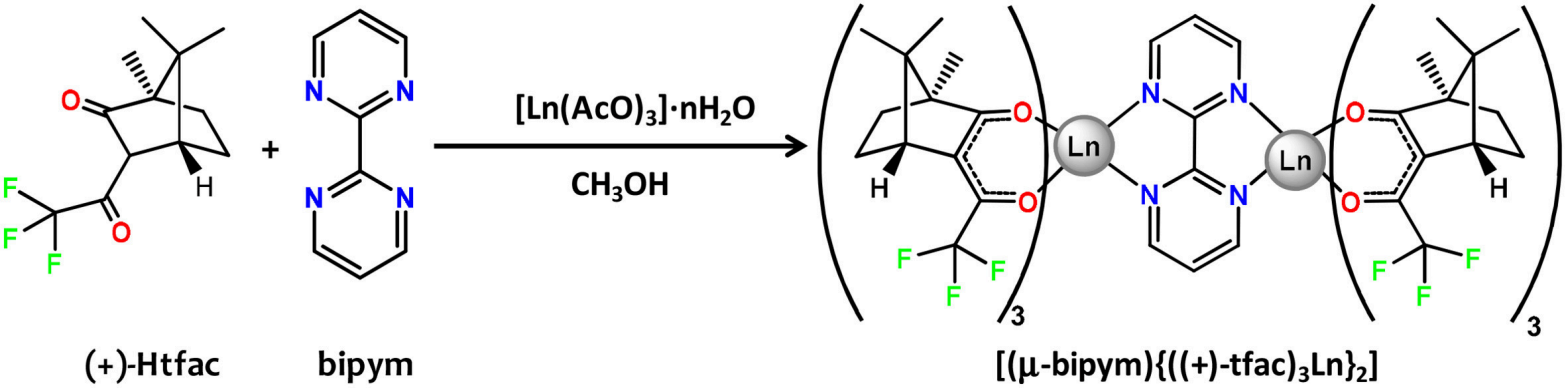
Syntheses of complexes d-**1/**1-**l** and d-**2**.

Complexes d-**1**, l-**1** and the GdIII counterpart d-**2** were prepared in one step by reacting 2,2′-bipyrimidine, Ln(AcO)_3_·nH_2_O (Ln^III^ = Dy, Gd) and the corresponding 3-trifluoroacetyl)camphor enantiomer in 1:2:6 molar ratio and using methanol as solvent (Scheme 1).

**Figure 1:**
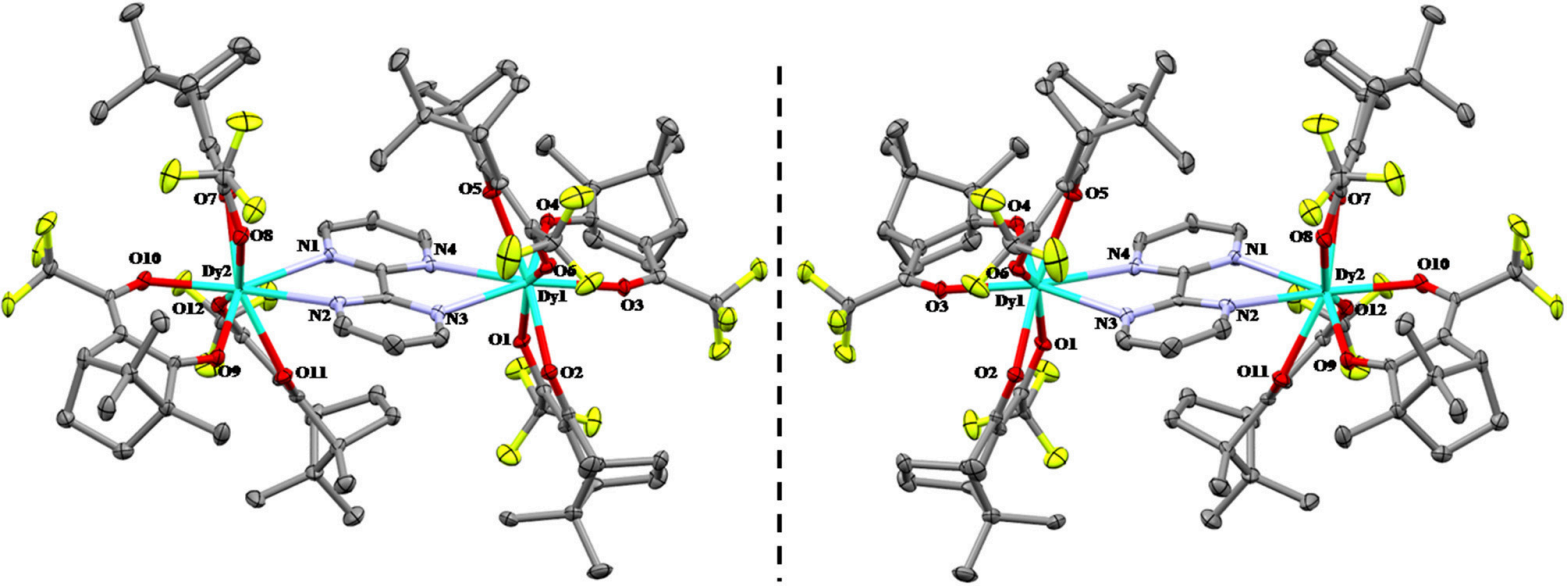
Crystal Structure of the enantiomeric pair l-**1 (Left)** and d-**1 (Right)**. Hydrogen atoms are omitted clarity. Ellipsoids are drawn at 50% probability.

The dinuclear complexes d-**1**, l-**1**, and d-**2** are, as expected, isostructural and crystallize in the triclinic polar P1 space group. We are going to describe below the structure of d-**1** as an example of all of them. The structure consists of well-isolated [(μ-bipym){((+)-tfacam)_3_Dy}_2_], in which two [Dy(tfacam)_3_] moieties are connected by the symmetric bis-bidentate 2,2′-bipyrimidine ligand, with an intramolecular Dy···Dy distance of 6.847 Å (Figure 1). Selected bond distances and angles are gathered on Tables S2, S3. Although both Dy^III^ ions exhibit DyN_2_O_6_ coordination environments, which are formed by the coordination of six oxygen atoms from the three tfacam^−^ diketonate ligands and the two nitrogen atoms from the bipym bridging ligand, however, they are crystallographically non-equivalent and show somewhat different bond distances and angles. The analysis of the DyN_2_O_6_ coordination spheres of Dy1 and Dy2 by the continuous-shape-measures (CShMs) method and SHAPE software (Llunell et al., 2005) reveals that their geometries are intermediate between several ideal eight-coordinated polyhedra. In the case of Dy1 the lowest CShMs parameters are 0.686 (D_4d_square antiprism) and 2.315 (D_2d_ triangular dodecahedron), whereas for Dy2 the lowest CShMs parameters are 1.083 (D_4d_square antiprism) and 1.085 (D_2d_ triangular dodecahedron), (Table S4). Therefore, for Dy1 the geometry is close to square antiprism, whereas the geometry of Dy2 is the is in between the D_4d_↔D_2d_ deformation pathway. The Dy-O_tfacam_ bond distances, which are found in the 2.272(4) Å (Dy1-O1)-2.377(4) Å (Dy2-O7) range, are shorter than the Dy-N_bipym_ distances, which fall in the 2.611(5) ((Dy2-N1) Å-2.621(3) Å (Dy1-N4) range. This fact is not unexpected, as the electrostatic interactions between the Dy^III^ ion and oxygen atoms of the tfacam^−^ ligands are larger than those involving the nitrogen atoms of the bipym bridging ligand. Similar bond distances have been observed for the analogous achiral [(μ-bipym){(β-diketonate)_3_Dy}_2_] complexes (Sun et al., 2016; Yu et al., 2016). The [(μ-bipym){((+)-tfacam)_3_Dy}_2_] molecules are well-isolated in the structure because they are neither involved in hydrogen bond interactions nor in close intermolecular contacts. The shortest Dy···Dy distance being of 9.496 Å.

**Figure 2:**
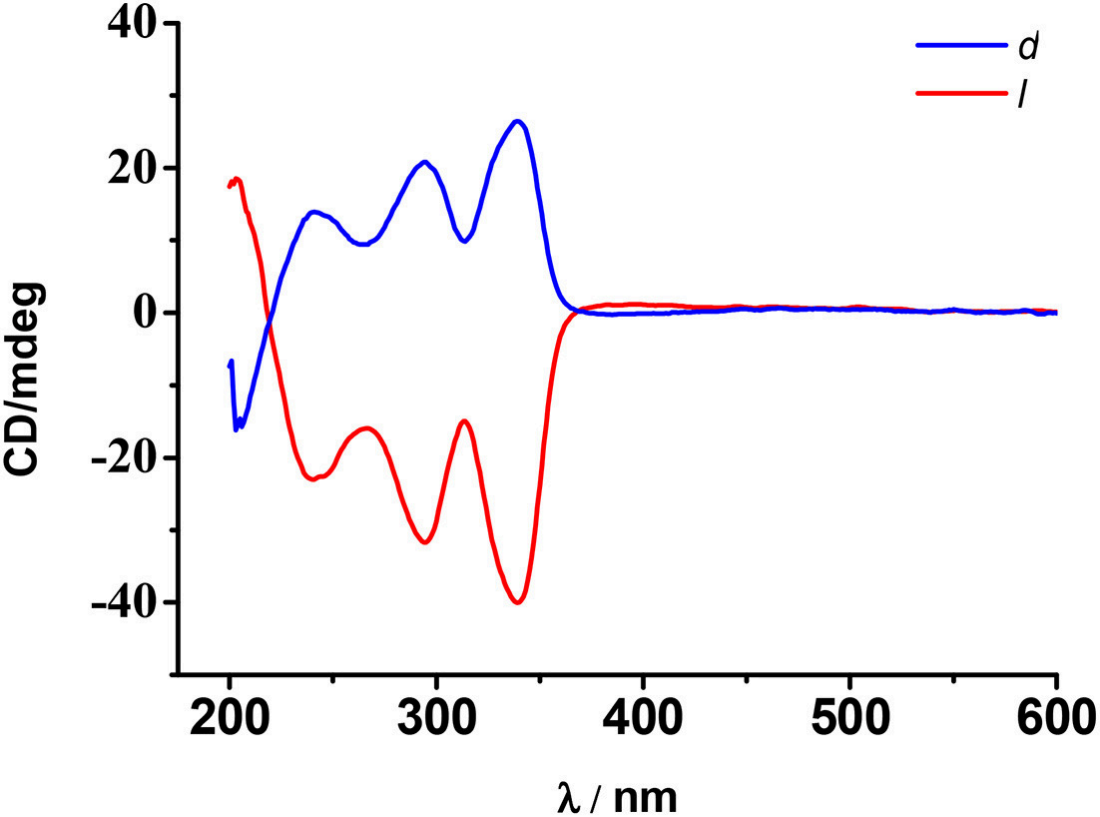
Solid-state CD spectra of d-**1**(blue)/l-**1**(red) based on a pressed KBr pellet of the respective crystals (1% wt) at room temperature.

The solid-state CD spectra of complexes d-**1** and l-**1** support their enantiomeric nature (Figure 2) as they exhibit almost mirror-image CD spectra. Cotton effects were observed for both d-**1**/l-**1** enantiomers between 200 and 350 nm. The spectrum of d-**1** shows positive Cotton effects at λmax = 240, 293 y 340 nm, whereas l-**1** shows Cotton effects with opposite signs at the same wavelengths. These results show that the chirality has been successfully transmitted from the ligand to the coordination environment of the Dy centers.

In spite of belonging to a P1 polar group, polarization measurements as a function of the applied electric field carried out on crystal samples of d-**1** and l-**1** at room temperature indicate that these compounds do not present P-E hysteresis loop and consequently ferroelectricity, exhibiting typical dielectric behavior. This fact could be due to the lack of enough charge separation between the Dy^III^ cation and the donor atoms of the ligands as to produce large electric dipolar moments.

### Magnetic properties

The magnetic properties are identical for both enantiomer, so we will described only the data obtained from d-enantiomers in this section.

**Figure 3:**
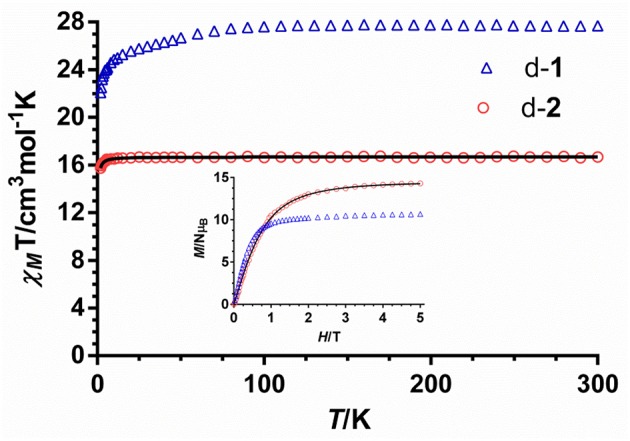
Temperature dependence of the χ_M_T product for compounds d-**1** and d-**2**. The solid line represents the best fit of the experimental data. Field dependence of the magnetization for d-**1** and d-**2** (inset).

The temperature dependence of the χ_M_T product for complexes [(μ-bipym){((+)-tfacam)_3_Dy}_2_](d-**1**) and [(μ-bipym){((+)-tfacam)_3_Gd}_2_](d-**2**) (χ_M_ being the molar magnetic susceptibility per dinuclear Ln^III^ unit) in the 2-300 K temperature range was measured with an applied magnetic field of 0.1 T (Figure 3).

At room temperature, the observed χ_M_T value for d-**1** (27.77 cm^3^·K·mol^−1^) is close to that expected for two independent Dy^III^ ions in the free-ion approximation (28.34 cm^3^·K·mol^−1^). Lowering temperature, the χ_M_T product remains almost constant down to 100 K, when it decreases steadily until approximately 20 K and then more sharply to reach a value of de 21.91 cm^3^·K·mol^−1^ at 2 K. This behavior is mainly due to the combined effects of the depopulation of the *M*_J_ sublevels of the Dy^III^ ions, which arise from the splitting of the ground term by the ligand field, as well as possible dipolar and exchange antiferromagnetic interactions. The magnetization vs. field plots for d-**1** at T = 2 K (Figure 3, inset) exhibit a fast increase of the magnetization up to ~ 1 T and then a slow increase with the field without reaching saturation at 5 T, which is mainly due to the presence of significant magnetic anisotropy and the possible existence of thermally and field accessible excited states. The magnetization value at the highest applied dc magnetic field of 5 T (10.7 N_μ*B*_) is about half of that calculated for non-interacting Dy^III^ ions (20 Nμ_B_), which can be mainly attributed to crystal-field effects giving rise to significant magnetic anisotropy and to an axial *M*_J_ = ± 15/2 ground state (Bi et al., 2011; Feltham et al., 2011; Ruiz et al., 2012). With the aim of analyzing the sign and magnitude of the magnetic interaction between the Dy···Dy ions in d-**1**, we have studied the magnetic properties of the isostructural Gd^III^ complex (d-**2**). We have followed this strategy because isostructural Gd^III^ and Dy^III^ containing complexes, generally, display the same type of magnetic coupling (Colacio et al., 2010). Nevertheless, this assumption should be taken with caution as some dinuclear Dy^III^ complexes with either oxalate or diphenoxide bridging groups exhibit ferromagnetic interactions between the Dy^III^ (Xu et al., 2010), whereas dinuclear Gd^III^ complexes containing such type of bridging ligands typically exhibit antiferromagnetic interaction between Gd^III^ ions. The room temperature χ_M_T value for d-**2** (16.68 cm^3^·K·mol^−1^) is close to the calculated value of (15.75 cm^3^·mol^−1^·K) for two magnetically independent Gd^III^ ions (4f^7^, S = 7/2, g_J_ = 2). On cooling down, the χ_M_T product remains constant down to 10 K and then decreases to reach a value of 15.73 cm^3^·K·mol^−1^ at 2 K. This behavior is more likely due to the combined action of very weak intramolecular exchange interactions between the Gd^III^ ions through the bipym bridging ligand, Zeeman saturation effects and very tiny ZFS of the ground state, which sometimes is observed for this essentially isotropic ion. The field dependence of the magnetization at 2 K (Figure 3, inset) shows a relatively rapid increase of the magnetization up to 2 T and then a linear increase to reach a value of 14.29 Nμ_B_ at 5 T, which is very close to the theoretical saturation value for two Gd^III^ ions with g = 2.0 (14 Nμ_B_).

The magnetic susceptibility and magnetization data of d-**2** were analyzed with the following Hamiltonian:

$$\begin{array}{l} H=\;-JS_{Gd1}S_{Gd2}+\left(g\mu_{B}S_{Gd1}+g\mu_{B}S_{Gd2}\right)B \end{array}$$

where the first term takes into account the intramolecular isotropic interaction between the Gd^III^ ions through the bipym ligand and the second term corresponds to the Zeeman effect (the g factor has been considered to be the same for the two Gd^III^ ions), μ_B_ is the Bohr magneton and B the applied magnetic field. The simultaneous best fit of susceptibility and magnetization data with the PHI software (Chilton et al., 2013a) afforded the following set of parameters:

*J* = −0.011 cm^−1^, *g* = 2.056 and *R* = 4·10^−4^ ($R=\sum {\left(\chi_{M}T_{\exp}-\chi_{M}T_{calc}\right)}^{2}/{\left(\chi_{M}T_{\exp}\right)}^{2}$). The extracted *J* value is of the same order of magnitude, but twice larger than that found in the analogous compound [(Gd(tmh)_3_)_2_(μ-bpym)] (*J* = −0.006 cm^−1^) and smaller than those extracted for other bipyrimidine-bridged Gd^III^ complexes containing different ancillary ligands with *J* values between −0.039 cm^−1^ and 0.053 cm^−1^ (Znovjyaka et al., 2009; Visinescu et al., 2010). These results show the non-innocent role played by the coligands in determining the magnitude of the magnetic exchange interactions in bipyrimidine-bridged Gd^III^ complexes. It should be noted that the extracted *J* value could be considered as the upper limit for the magnetic interaction because it must also comprise the effects of the possible ZFS and Zeeman interaction. In view of these results, it would be reasonable to assume that d-**1** also exhibits antiferromagnetic interaction between the Dy^III^ ions.

**Figure 4:**
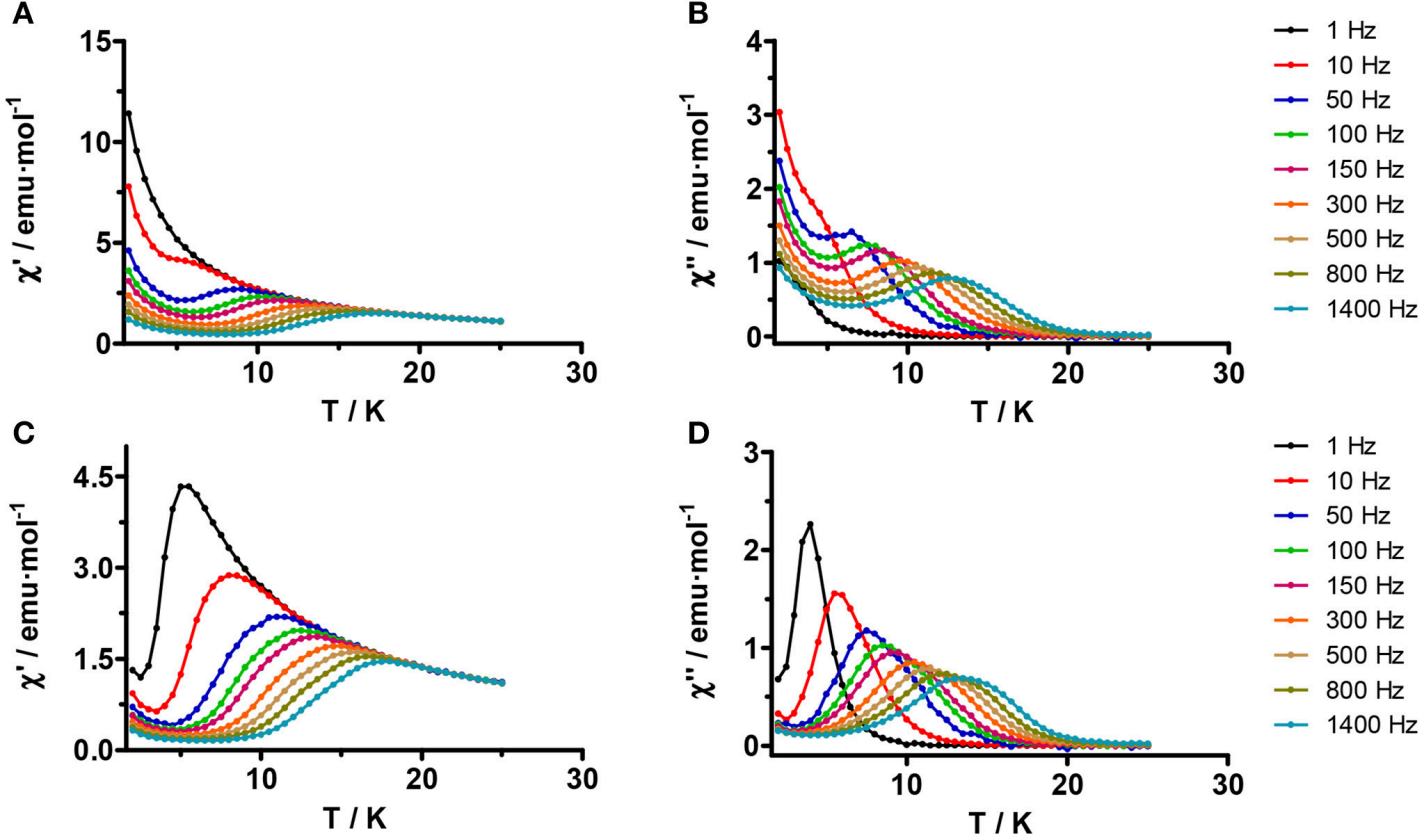
Temperature dependence of the in-phase **(A,C)** and out-of-phase **(B,D)** *ac* susceptibility signals at zero **(A,B)** and 0.1 T **(C, D)** for complex d-**1**. Solid lines are only a guide for the eye.

The ground Kramers doublet of the Dy^III^ ion in d-**1** is axial (see below and *ab initio* calculations) and, in principle, it should exhibit SMM behavior. In good agreement with this, complex d-**1** shows frequency and temperature dependence of the out-of-phase magnetic susceptibility (χ”_M_) at zero field below 20 K (Figure 4) with maxima in the 12.5 K (1,400 Hz)−6.5 K (50 Hz) temperature range.

**Figure 5:**
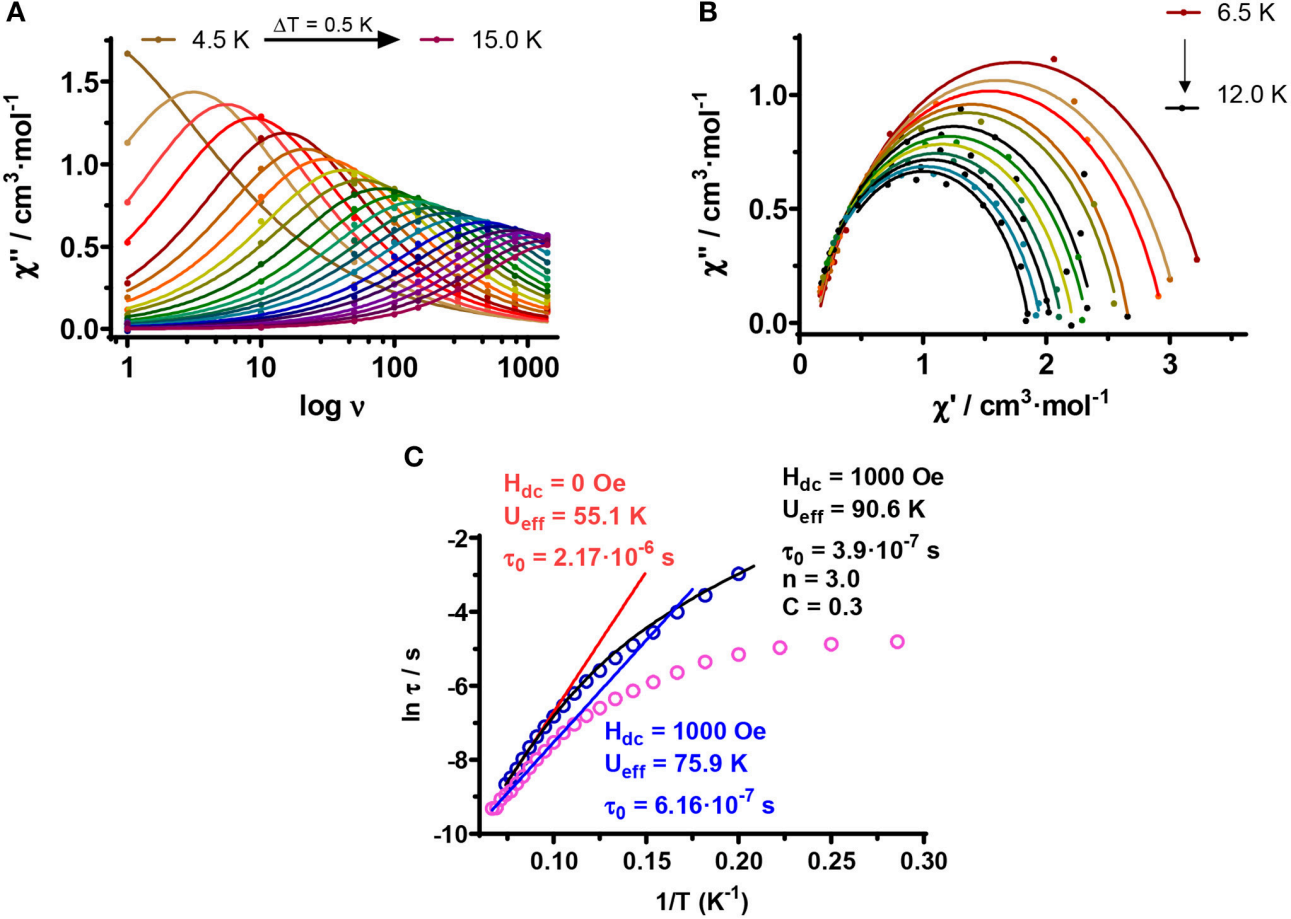
**(A)** Frequency dependence of out-of-phase *ac* susceptibility signals at 0.1 T (solid lines represent the best fit to the Debye model), **(B)** Cole-Cole plot, and **(C)** Temperature dependence of the relaxation times for complex d-**1**. Blue and red solid lines correspond to the Arrhenius plots for data at zero and 0.1 T, respectively. The black solid line represents the best fit of the temperature dependence of the relaxation times at 0.1 T to a combination of Orbach and Raman relaxation processes.

This behavior is indicative of slow relaxation of the magnetization and confirms its SMM behavior. Moreover, χ“_M_ shows a relatively strong increase (Figures 4A,B) below 4 K, which could be due to fast QTM relaxation. It is worth noting that in spite of the fact that the Dy centers are non-crystallographically equivalent, the temperature, and frequency dependence of the χ”_M_ signals do not show two maxima but instead a broad peak, thus indicating that the relaxation processes for the two Dy centers have very close thermal energy barriers. The temperature dependence of the relaxation times for magnetization reversal (τ) was extracted from the fit of the frequency dependence of χ“_M_ at different temperatures to the generalized Debye model. Fitting the extracted relaxation times to the Arrhenius equation in the high temperature region (10–15 K), afforded an effective energy barrier for the reversal of the magnetization *U*_eff_ = 55.1 K and a pre-exponential factor τ_o_ = 2.17·10^−6^ s (Figure 5C). The τ_o_ value is larger than those usually found for Dy^III^ based SMMs, which can be due to the existence of QTM. As it can be observed in the Arrhenius plot, the relaxation times deviate from the linearity below 10 K and became almost temperature independent below 4 K, which allows us to extrapolate a relaxation time for the QTM process τ_QTM_ = 8 μs. The Cole-Cole plots show semicircular shapes with α values in the 0.343 (5.0 K)−0.164 (13.0 K) region, thus confirming the presence of a distribution of relaxation processes (Figure S9). The α parameter determines the width of the distribution of relaxation times, so that α = 1 corresponds to an infinitely wide distribution of relaxation times, whereas α = 0 represents a process with only a single relaxation time.

It is well-known that QTM in the ground state can be promoted by transverse anisotropy, as well as dipolar and hyperfine interactions. In order to suppress QTM, two strategies are generally used: (i) to apply a small dc field, which removes degeneration in the ground and excited states thus preventing, at least partly, QTM (ii) Magnetic dilution with an isostructural diamagnetic compound for declining dipolar interactions. In view of this, we decided to prepare an isostructural diluted compound of d-**1** containing a Dy^III^/Y^III^ = 1/20 molar ratio (namely d-**1'**) and to measure the temperature dependence of the ac susceptibilitity at zero field and 0.1 T (this is the optimal field inducing a larger relaxation time, Figure S10), as well as that of the undiluted compound at 0.1 T, using a frequency of 1,400 Hz (Figure S11). From this study the following conclusions can be drawn: (i) Peaks at 0.1 T appear slightly shifted to higher temperatures than those at zero field for the undiluted as well as for the diluted compound. (ii) The low temperature tail due to QTM almost disappears for the diluted complex as well as when a dc field of 0.1 T is applied on the undiluted compound. In view of this, we decided to perform a complete set of ac measurements in the presence of a *dc* magnetic field of 0.1 T.

As it can be observed in Figures 4C,D, the maxima in the χ_M_” vs T plot now appear between 13.5 K (1,400 Hz) and 4.0 K (1 Hz). Moreover, the χ_M_” signal tends to zero after the maxima, thus pointing out that the QMT has been almost completely eliminated. Fitting of the frequency dependence of χ”_M_ at different temperatures to the generalized Debye model, afforded the relaxation times of the magnetization (τ) at each temperature (Figure 5A). The effective energy barrier for the reversal of the magnetization (*U*_eff_) and the pre-exponential factor τ_0_ extracted from the fit of τ in the high temperature region (15–10 K) to an Arrhenius law were: *U*_eff_ = 75.9 K and τ_o_ = 6.16 × 10^−7^ s (Figure 5C). As expected, the application of a small *dc* field of 0.1 T to eliminate, at least partly, the fast relaxation due to QTM, generally induces a slow down of the magnetization relaxation with a concomitant increase of *U*_eff_ (from 55.1 to 75.9 K) and a decrease in τ_0_.

The deviation of the data from the Arrhenius law below 10 K is a clear indication of the coexistence of several competing relaxation processes. The Cole-Cole plots for these complexes show semicircular shapes in the 6.5–12 K temperature range (Figure 5B) with α values ranging between 0.211(6.5 K) and 0.158(12 K) which support the existence of several relaxation processes. Since in the studied temperature range (*T* > 6.5 K and 0.1 T) direct and QTM relaxation processes should be almost negligible, we have fitted the magnetic data to Equation (1), which considers that Raman and Orbach processes contribute simultaneously to the relaxation of the magnetization.

(1) $$\begin{array}{r} \tau^{-1}={\text{CT}}^{\text{n}}+\tau_{0}^{-1}\exp\left(-U_{eff}/k_{B}T\right) \end{array}$$

The extracted parameters were: C = 0.31 s^−1^K^*n*^, *n* = 3.0, *U*_eff_ = 90.6 K and τ_o_ = 3.9 × 10^−7^s. Although *n* = 9 is expected for Kramers ions (Abragam and Bleaney, 1970), this may change depending on the structure of the levels and if both, acoustic and optical phonons, are considered. Thus *n* values between 1 and 6 are considered as acceptable (Singh and Shrivastava, 1979; Shirivastava, 1983). As usual, *U*_eff_ is higher than that obtained at 0. 1 T from the simple Arrhenius law, while the pre-exponential factor τ_0_ decreases by half.

It should be noted at this point that the value of χ_M_'T (χ_M_' is the in-phase ac susceptibility, Figure S12) at its low temperature plateau (where all the lines are coincident) of 25.2 cm^3^mol^−1^ K agrees well with the value expected for randomly oriented crystals with a *M*j = ±15/2 Ising ground Kramers doublet (25 cm^3^ mol^−1^ K).

**Figure 6:**
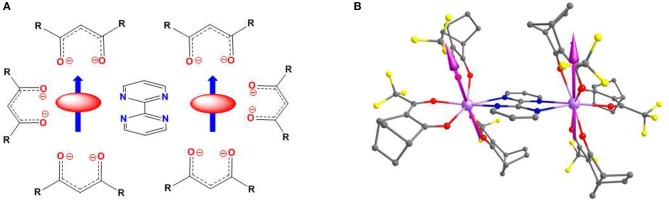
**(A)** Left Qualitative prediction of the orientation of the anisotropic axes (blue arrows) based on the oblate-prolate electrostatic model and **(B)** Right Computed anisotropy axis of both Dy centers. Hydrogens are omitted for clarity. Color code: C-Gray, N-Blue, O-Red, Cl-Yellow Dy-Violet.

The Dy-O bond distances are shorter than the Dy-N ones in the DyN_2_O_6_ coordination spheres of Dy1 and Dy2, and therefore the former have larger electron density than the latter. Taking into account this, the two tfacam^−^ ligands at opposite sides of each Dy atom, create an appropriate axial crystal field with the remaining positions in the equatorial plane occupied by the oxygen atoms of the other tfacam^−^ bidentate ligand and the N,N atoms belonging to the neutral bipym bridging ligand. In this disposition, as qualitatively predicts the simple electrostatic oblate-prolate model (Rinehart and Long, 2011), the oblate electron density of the Dy^III^ ion is forced to be located in the equatorial plane, thus diminishing the electrostatic repulsions with the oxygen atoms of the two β-diketonato ligands defining the axial crystal field (Figure 6A). If so, the anisotropy axis should be located close to the two tfacam^−^ligands at opposite sides of each Dy^III^ atom. We have calculated the direction of the anisotropy axes of the Dy^III^ ions by using the Chilton's method (Chilton et al., 2013b), which is based on electrostatic arguments, and the results (see Figure 6B) confirm our qualitative prediction using the prolate-oblate model, with an angle between the anisotropy axes of 2.9°.

In order to know how the structural parameters of the DyN_2_O_6_ coordination sphere affect the SMM behavior of d-**1** and their achiral [(Dy(β-dicetona)_3_)_2_(μ-bpym)] analogs, the magneto-structural data for these compounds have been compared in Table 1.

**Table 1:** Magneto-structural data for [(Dy(β-dicetona)_3_)_2_(μ-bpym)] complexes.

**Complex**	**Experimental blocking barrier U_eff_ (cm^−1^)**	***Ab initio*** **predicted blocking barrier U**_**cal**_ **(cm**^**−1**^**)**		**CShM parameter**^**a**^ **(SAPR-8)**		**CShM parameter**^**a**^ **(TDD-8)**		**References**
		**Dy1**	**Dy2**	**Dy1**	**Dy2**	**Dy1**	**Dy2**
d-1	55.1	141.97	129.95	0.686	1.083	2.315	1.085	This work
2	201	183.87	157.48	1.545	0.947	0.846	1.510	Sun et al., 2016
	67						
3	267	257.83	257.83	1.630	1.630	0.888	0.888	Sun et al., 2016
4	97	133.70	133.93	0.605	0.605	2.375	2.375	Yu et al., 2016

a *CShM, Continuous shape measurements*.

As it can be observed in Table 1, it seems that the Dy^III^ ions with a geometry much closer to trigonal dodecahedron D_2d_ (that having the lowest continuous shape measurement for this geometry) exhibit, in general, higher *U*_eff_ values. Taking into account this correlation, the *U*_eff_ for compound d-**1** should be similar, or even higher, as that for compound [(Dy(tmh)_3_)_2_(μ-bpym)] (**4**). Surprisingly, the experimental *U*_eff_ value for the latter compound is higher. Theoretical *ab initio* calculations have been carried out on **1**–**4** to justify this discrepancy.

## Theoretical calculations

*Ab initio* calculations on the d-**1** and the previously reported achiral analogous were undertaken using the CASSCF+RASSI-SO method. The aim of this study is two-fold: (i) To support the presence of axial anisotropy and to shed light on the mechanism of the slow magnetic relaxation of these compounds (ii) To confirm the above indicated magneto-structural correlation and to justify the deviation of d-**1** from it.

**Figure 7:**
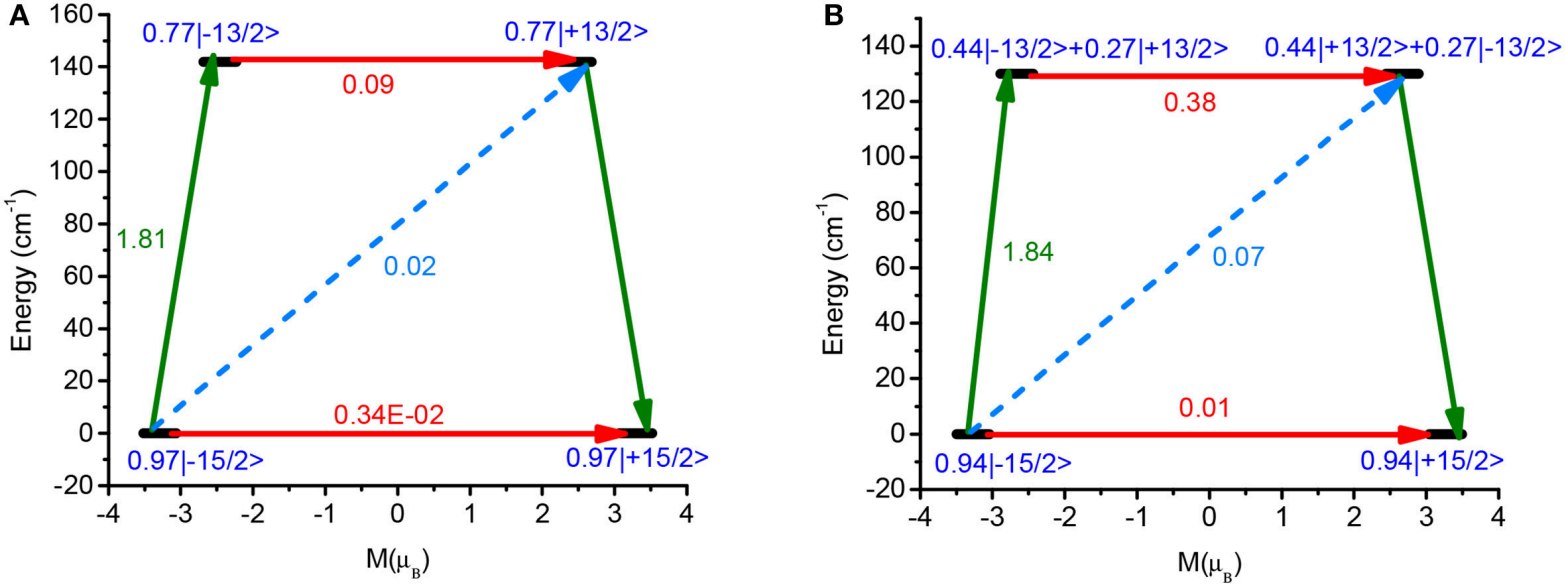
**(A)** Relaxation mechanism of the Dy1 center in d-**1**. **(B)** Relaxation mechanism of the Dy2 center in d-**1**.The Black line indicates the KDs as function of magnetic moments. The red line represents QTM via ground states and TA-QTM via excited states. Dashed line indicates possible Orbach process.

The calculation on d-**1** has been performed using the X-ray crystal structure where methyl group has been modeled by hydrogen to reduce the computational cost. The eight Kramers doublets (KDs) generated from ^6^H_15/2_ spin orbit coupled ground term span up to 538 cm^−1^ for Dy1 (540 cm^−1^ for Dy2) suggesting moderately axial ligand field generated in the D_4d_ environment. The computed anisotropy axis of both the centers is shown in Figure 6B. The g_zz_ axes of both centers are collinear with a tilt angle of just 3.8°, which is very close to that found using the electrostatic model (see above). The energy spectrum, g tensors, angle of the excited state g_zz_ with the ground state of both Dy centers is listed in Tables 2, 3.The g_zz_ axis of both the Dy^III^ centers is found to lie along the β-diketonato ligands parallel to each other to mimic to axial crystal field. The equatorial positions are occupied by the two nitrogen atoms of the of the bipyrimidine ligand and two oxygen atoms of a β-diketonato ligand thus minimizing, as indicated elsewhere (Figure 6A), the electrostatic repulsion between the oblate electron density of Dy^III^ ions with the above mentioned atoms in the equatorial plane. The ground state of both centers is of Ising type as the transverse components of anisotropy in this state are very small and the magnetic moment g_z_ reaches a value close to the value generated from pure m_J_ = 15/2 state (g_z_ = 20). Although the two centre contains significant amount of transverse anisotropy in the KD2, the magnitude is larger for the Dy2 centre. The axiality of both centers started to decrease as it goes to higher KDs while it becomes lowest at 4th excited state, then it started to increase and becomes maximum at KD8. The relaxation mechanisms of both centers are shown in Figure 7. The energy gap between the ground and the first excited state is 141.97 and 129.95 cm^−1^ for Dy1 and Dy2 center respectively. This implies that the two Dy centers resides in different structural environments. The QTM and TA-QTM of the Dy1 center is estimated to be ten times less compare to Dy2 center. This is reflected by their corresponding wave function analysis of the KDs. The ground state composition of the |15/2> state of the Dy1 center becomes larger compare to Dy2 center. The major contribution of the first excited KDs comes from the |13/2> state where the mixing of this state with other states becomes larger for Dy2 center which increase the TA-QTM for this center. The gzz axis of the ground and first excited state are nearly collinear, thus suggesting that relaxation is likely to proceed further via higher excited states, however very large value of TA-QTM and transverse anisotropy present in the first excited state enforce relaxation via this state. The probability of Orbach (1.81/1.84) and Raman process (0.02/0.07) remain almost same for both the centers while the Raman relaxation is found to be very small. The larger QTM and TA-QTM of the Dy2 center compared to the Dy1 center can be explained by their corresponding distortion from the D_4d_ (SAPR-8) geometry. The larger deviation of the Dy2 center (1.083) compare to Dy1 center (0.683) in square antiprism geometry facilitate the larger QTM and TA-QTM in the former center.

**Table 2:** Calculated g tensors with their corresponding energy spectrum, angle of the anisotropy axis of the excited states with the corresponding ground state (°) of the Dy1 center in d-**1**.

**Energy (cm^−1^)**	**g_x_**	**g_y_**	**g_z_**	**Angle of g_zz_ between ground and higher excited KDs(^°^)**
0.00	0.007	0.012	19.761	–
141.97	0.162	0.357	16.240	6.34
213.15	0.665	2.619	17.106	68.52
224.90	0.281	1.266	12.155	37.66
268.92	4.550	5.701	7.508	77.77
304.30	1.165	2.287	16.545	64.05
436.37	0.063	0.210	17.125	108.74
538.14	0.043	0.111	18.789	123.80

**Table 3:** Calculated g tensors with their corresponding energy spectrum, angle of the anisotropy axis of the excited states with the corresponding ground state (°) of the Dy2 center in d-**1**.

**Energy (cm^−1^)**	**g_x_**	**g_y_**	**g_z_**	**Angle of g_zz_ between ground and higher excited KDs(^°^)**
0.00	0.025	0.044	19.61	–
129.95	0.706	1.476	15.312	5.13
187.25	2.350	3.806	13.155	50.01
220.57	1.919	3.895	11.461	60.60
257.15	2.331	2.982	13.955	91.27
306.81	0.148	0.188	19.232	62.85
395.09	0.084	0.136	18.184	107.03
540.66	0.013	0.023	19.501	124.65

The *ab initio* calculated effective energy barrier for Dy1 and Dy2 centers (141.97 and 129.95 cm^−1^) are overestimated compared to the experimental U_eff_ value (55.1 cm^−1^) for magnetization reversal. The discrepancy essentially arise from the (i) QTM which is operative in zero field prompted by dipolar and hyperfine interactions reduces the barrier height. (ii) The limitations computational methodology used (absence of dynamic correlation, modeled structure etc).

The deviation can be explained from the *ab initio* calculated crystal field parameter. The crystal field parameters are computed (Table S11) using the following equation as implemented in SINGLE_ANISO code ${Ĥ}_{CF}=\;\sum \overset{q}{\underset{k=-q}{\sum }}B_{k}^{q}{Õ}_{k}^{q}$, Where ${Õ}_{k}^{q}$ and $B_{k}^{q}$ are the computed extended Stevens operator and crystal field (CF) parameter, respectively. The probability for the occurrence of QTM is higher when the non-axial $B_{k}^{q}$ terms (q≠0 and *k* = 2, 4, 6) are larger than the axial (q = 0 and k = 2, 4, 6) terms. The negative value ${\text{B}}_{2}^{0}$ parameter corroborates the stabilization of |15/2> as ground state. The computed non-axial crystal field parameters (${\text{B}}_{2}^{2}$, ${\text{B}}_{2}^{1}$, ${\text{B}}_{2}^{-1}$) are larger than the axial parameters in d-**1** which facilitates the QTM in zero field and explains the overestimation of U_cal_.

In order to gain a deeper insight into the role of magnetic exchange to magnetic relaxation POLY_ANISO program was employed which interfaced with SINGLE_ANISO of individual Dy centers. The calculated g tensors of both the Dy centers strongly anisotropic imply that the resulting interaction is strongly anisotropic. The experimental magnetic susceptibility was fitted by Lines approach (Lines, 1971) to estimate intramolecular and dipolar interactions between Dy^III^-Dy^III^. This methodology has been shown to yield good numerical estimate of *J*s when compared to the experimental values in a variety of dinuclear systems (Oyarzabal et al., 2015; Gupta et al., 2016b; Singh et al., 2017).

**Figure 8:**
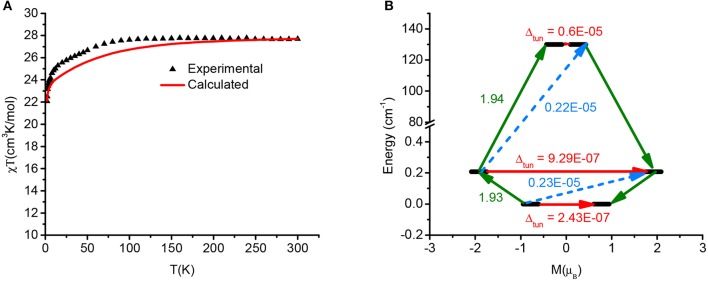
**(A)** Fitted experimental magnetic susceptibility using Lines model and **(B)** POLY_ANISO computed blockade barrier of d-**1**. The black lines indicate exchange states that have been arranged in compliance with the value of its magnetic moment. The red arrows (and pertinent values) correspond to tunneling transitions within ground-state and first excited-state exchange doublets. However, olive and dashed arrows and their corresponding values represent transition magnetic moment matrix elements of spin-phonon relaxation pathways.

The magnetic interactions is modeled by H = –*J*s_1z1_s_2z2_, where s_1z1_ and s_2z2_ are the projections of the effective spin $\tilde{s}$ = 1/2 of the lowest KDs of the Dy^III^ ions on the principal anisotropy axes. A good fit is obtained by considering *J*_tot_ = 0.0039 cm^−1^ (*J*_exch_ = 0.004 cm^−1^ and *J*_dipolar_ = −0.0001 cm^−1^) (Figure 8).

The magnetic anisotropy axis of both Dy^III^ centers are nearly parallel to each other and this is compatible with the ferromagnetic interaction obtained from Lines model. The significant tunnel splitting (0.6^*^10–5) in the third exchange doublets blocks the relaxation via this state (see Table S16 for the spectrum of lowest exchange levels). The observed magnetization blockade of 131 cm^−1^is very similar to that forum in the single ion analysis. This is due to the expected weaker exchange coupling between Dy ions. Therefore, the experimentally observed barrier height arises from the contribution of individual Dy^III^ fragments.

We have undertaken a comparative study to elucidate the role of the distortion parameter from the corresponding symmetry to the magnetic anisotropy of the metal center. We have chosen three Dy_2_ complexes analogous to d-**1** containing bipyrimidine as bridging ligand, six β-diketones as end-cap ligands and a similar DyN_2_O_6_ core environment: [(Dy(DBM)_3_)_2_(μ-bpym)].2CH_3_Cl(**2**), [(Dy(DBM)_3_)_2_(μ-bpym)]MeCN (**3**), and [(Dy(tmh)_3_)_2_(μ-bpym)] (**4**). The first two molecules has been synthesized by Song Gao et. al. (Sun et al., 2016) and the third molecule has been studied by Mario Ruben et al. (Yu et al., 2016). The CShM values of the above three molecules along with those of d-**1** are listed in the Table 1. All the three complexes exhibit SMM behavior in zero field with the effective energy barriers indicated in Table **1**. The detailed results of the theoretical study carried out on the X-Ray crystal Structures of **2**-**4** are given in the ESI (Tables S5–S10, S12–S15, S17–S19 and Figures S1–S7).

The calculated blocking barrier as well the angle between the anisotropy axis of each Dy^III^ center with the Dy1-Dy2 axis and the tilt angle between the anisotropy axes of both Dy^III^ centers are given in Tables 1, 4, respectively (the rest of computed parameters, like to those extracted for 1, are given in the ESI).

**Table 4:** Angle of anisotropy axis of each Dy^III^ center with the Dy1-Dy2 axis and the tilt angle between the anisotropy axes of both Dy^III^ centers.

**Complex**	**Angle (**^**°**^**)**		
	**g_zz1_-Dy1-Dy2**	**g_zz2_-Dy1-Dy2**	**g_zz1_-Dy1-Dy2- g_zz2_**
d-1	84.22	93.41	3.8
2	88.44	93.19	28.80
3	93.83	93.64	0.00
4	84.93	95.12	0.04

The results indicate that the a*b initio* computed barrier heights clearly follow the trend obtained from the distortion parameters. The study of the Dy1 center in complexes **1** and **4** reveals that the computed barrier height decreases from 141.97 cm^−1^ to 133.70 cm^−1^ as the CShM parameter in the TDD-8 geometry increases from 2.315 to 2.375. A close look on both Dy^III^ centers in complex d-**1** and **2** reveals that the barrier height decreases from 141.97 cm^−1^ to 129.95 cm^−1^ and from 183.87 cm^−1^ to 157.48 cm^−1^ as the CShM parameter increases from 0.686 to 1.083 and 0.846 to 1.510 in the SAPR-8 and TDD-8 geometry respectively for d-**1** and **2**.

A close look on the Table 4 reveals that as the g_zz1_-Dy1-Dy2 angle increases the computed barrier height of the Dy1 center increases (Angle; 84.22 → 88.44 → 93.83 → 84.93 barrier height; 141.97 → 183.87 → 257.83 → 133.70). This proportionality between the g_zz1_-Dy1-Dy2 and barrier height elucidate that the former angle should be kept as large as possible to get the large barrier height.

## Pulse magnetization measurements

**Figure 9:**
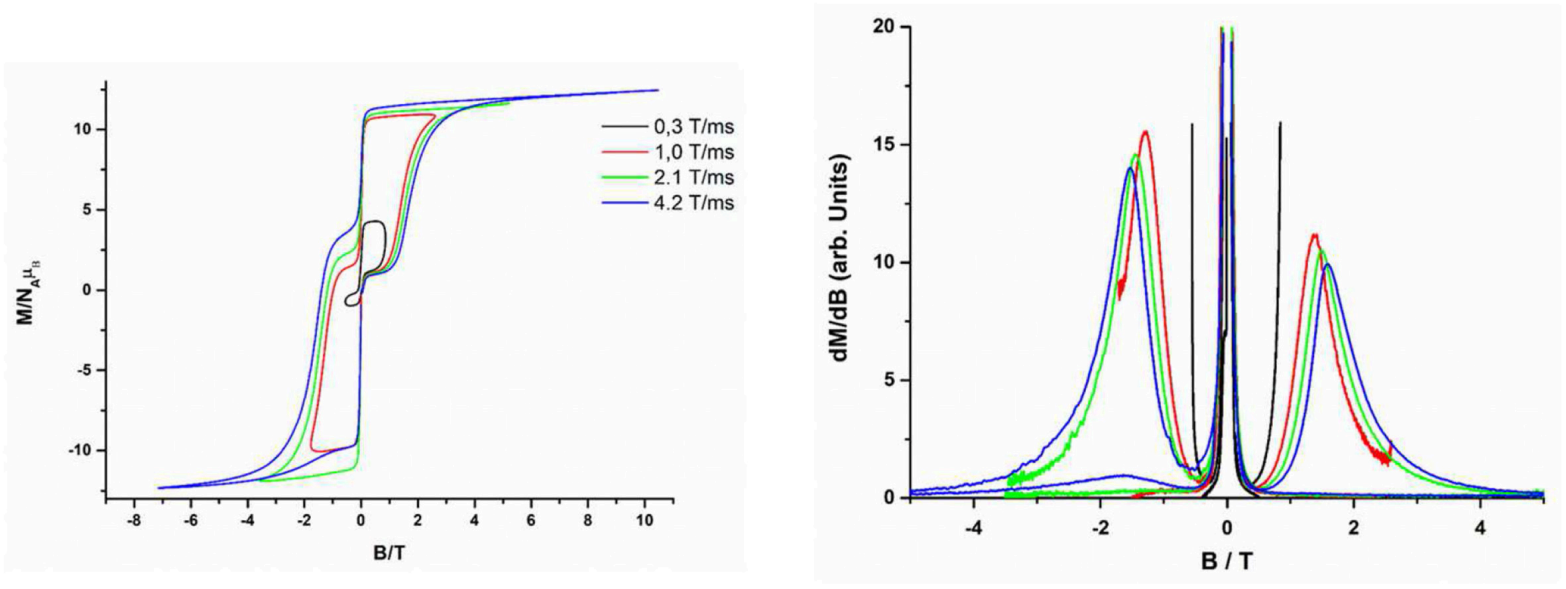
Pulsed-field magnetization curves at maximum fields of 0.84 T, 2.6 T, 5.2 T, and 10.4 T **(Left)** and differential of magnetization measured at 0.4 K **(Right)** for compound d-**1**.

The magnetization curves in a full cycle pulsed magnetic field at 0.4 K (Saito and Miyashita, 2001), with maximum fields of 0.84, 2.6, 5.2, and 10.4 T, were measured with the aim of confirming the SMM properties of d-**1** (Figure 9). The sweep rate depends on the maximum pulsed field, so it is the higher for 10.4 T (4.2 T/ms). Magnetization curves show large hysteresis loops, a sharp reversal at around zero field and saturations at high fields (except for the curve measured at a sweep rate of 0.3 T/s). The hysteresis increases with faster sweeping rate, which is characteristic of SMMs. The saturation moment per Dy^III^ ion is lower than the expected value (20 μβ/f.u.), which can be due to the misalignment between the local magnetization directions of Dy1 and Dy2 ions, as well imperfect alignment with the field. Moreover, the sharp reversal around zero points out that there is an adiabatic magnetization reversal most probably caused by QTM with a small tunneling gap. As indicated elsewhere, the gap may be caused by hyperfine interactions, weak inter-molecular interactions and/or transverse components of the magnetization by the low symmetry around Dy^III^ ion.

To analyse the origin of the loop, the differential magnetization dM/dB was plotted (Figure 9 right).

The presence of only one step in the dM/dB vs B plot of the dinuclear complex d-**1** at about 1.5 T (at positive as well as negative values of the field) could be due to either: (i) the direct relaxation between the ferromagnetic ground state (↓↑) and the excited antiferromagnetic state (↑↑) originated by magnetic coupling between two Dy ions or (ii) the almost equivalence between Dy1 and Dy2 when magnetic coupling through the bridging ligand is considered to be negligible.

## Conclusions

In summary, the assembly of 2,2′-bypirimidine, Ln(AcO)_3_·nH_2_O (Ln^III^ = Dy, Gd), and enantiomerically pure (+)/(−)-3-(trifluoroacetylcamphor) is revealed as a good strategy to prepare chiral bipyrimidine-bridged Ln^III^ complexes. The Dy^III^ counterparts possesses and almost pure axial ground Kramers doublet and SMM behavior at zero-field. The experimentally extracted anisotropy barrier for these Dy^III^ complexes is much lower than those obtained for other previously reported analogous complexes [(Dy_2_(β-dicetona)_3_)_2_(μ-bpym)], which is proposed to be due to the comparatively larger distortion for the former in the DyN_2_O_6_ coordination sphere from trigonal dodecahedron geometry. Theoretical *ab initio* calculations carried out on this [(Dy_2_(β-dicetona)_3_)_2_(μ-bpym)] complexes indicate that: (i) The calculated blocking barriers follow the above trend of smaller thermal energy barrier for magnetization reversal when the distortion from D_2d_ geometry of the DyN_2_O_6_ coordination sphere is larger. (ii) The relatively low *U*_eff_ value for the Dy^III^ complexes reported here is due to the existence of a comparatively larger QTM in the ground state. (iii) In contrast to the Gd^III^ complexes, which show weak antiferromagnetic interaction between the Gd^III^ ions, the Dy^III^ complexes, with one exception, exhibit very weak ferromagnetic interactions between the Dy^III^ ions. This fact clearly indicates the difficulty in accurately determining the sign of very weak magnetic exchange interactions. (iii) The experimentally observed barrier height mainly arises from the contribution of individual Dy^III^ fragments due to the weakness of the magnetic exchange interaction. (iv) Relaxation mechanisms for magnetization reversal justify the order of the experimentally extracted *U*_eff_ values.

Finally, pulse magnetization measurements show only one step in the field dependence of the dM/dB for the Dy^III^ complexes at about 1.5 T, which is due to either the direct relaxation between the ferromagnetic and antiferromagnetic states, originated by magnetic coupling between two Dy ions, or more likely to very weak coupling between equivalent Dy1 and Dy2 centers.

The reported results represent an additional example of how enatiomerically pure ligands can be successfully used to generate bifunctional SMM/chiral materials.

## Author contributions

ID-O prepared all the complexes, carried out their spectroscopic characterization, and made supplementary material. JH carried out the magnetic study and the analysis of the results. EC and GR wrote and revised this paper. JG-M and AR carried out ferroelectricity measurements and revised the paper. GR and SD performed theoretical *ab initio* calculations. HN performed pulse magnetization measurements.

### Conflict of interest statement

The authors declare that the research was conducted in the absence of any commercial or financial relationships that could be construed as a potential conflict of interest.
