# Supplementary material for: A Chiral Bipyrimidine-Bridged Dy2 SMM: A Comparative Experimental and Theoretical Study of the Correlation Between the Distortion of the DyO6N2 Coordination Sphere and the Anisotropy Barrier
Source: Front Chem. 2018 Nov 8;6:537. doi: 10.3389/fchem.2018.00537 (PMC6236069; doi:10.3389/fchem.2018.00537)
Supplement: Supplementary file 1 [file Data_Sheet_1.pdf]

<u>**Electronic Supporting Information**</u>

A Chiral Bipyrimidine-Bridged Dy_2 SMM: A Comparative Experimental and Theoretical study of the Relaxation Mechanism

Ismael F. Díaz-Ortega,[†] Juan Manuel Herrera,*[,†] José Ramón Galán-Mascarós,[⊥] Álvaro Reyes Carmona,[⊥] Sourav Dey,[#] Gopalan Rajaraman,*[,#] H. Nojiri,*[,‡] Enrique Colacio*[,†]

[†]*Departamento de Química Inorgánica, Facultad de Ciencias, Universidad de Granada, Avda. Fuentenueva s/n, 18071, Granada, Spain. ecolacio@ugr.es,* jmherrera@ugr.es

[⊥] *Catalan Institution for Research and Advanced Studies, PasseigLluisCompanys 23, Barcelona 08010, Spain*

[#]*Department of Chemistry, Indian Institute of Technology Bombay, Powai, Mumbai 400076, India.rajaraman@chem.iitb.ac.in*

[‡]*Institute for Materials Research, Tohoku University, Katahira, Sendai, 980-8577, Japan.*nojiri@imr.tohoku.ac.jp

Table S1. Crystallographic data for complexes d-1 and l-1.

	**d-1**	**l-1**
Formula	$C_{80}H_{90}Dy_2F_{18}N_4O_{12}$	$C_{80}H_{90}Dy_2F_{18}N_4O_{12}$
Mw (g·mol^{-1})	1966.55	1966.55
Crystal System	triclinic	triclinic
Space group	P1	P1
a (Å)	10.0697(7)	10.0837(6)
b (Å)	13.0130(10)	13.0136(7)
c (Å)	17.3895(13)	17.3873(9)
α (º)	101.678(2)	101.758(2)
β (º)	103.197(3)	103.256(2)
γ (º)	106.674(2)	106.578(2)
Z	1	1
D_c (g·cm^{-3})	1.604	1.602
M (Mo*Kα*) (mm^{-1})	1.924	1.922
T (K)	100	100
F(000)	988.0	988.0
2Θ range for data collection/°	4.434 to 56.696	5.016 to 49.836
Reflec. collected / unique	53025 / 18205	32344 / 12708
Data/restraints/parameters	18205/3/1063	12708/51/1063
GOF on F^2	1.081	1.044
$R_1^{a,b}$	0.0201(0.0181)	0.0254 (0.0214)
wR_2^c	0.0403 (0.0410)	0.0406 (0.0418)
Largest diff. peak/hole / e Å^{-3}	0.81/-0.80	0.34/-0.55
Flack parameter	0.001(3)	-0.029(4)

[a] $R_1 = \Sigma||F_o| - |F_c||/\Sigma|F_o|$. [b] Values in parentheses for reflections with $I > 2\sigma(I)$. [c] $wR_2 = \{\Sigma[w(F_o^2 - F_c^2)^2] / \Sigma[w(F_o^2)^2]\}^{1/2}$

Table S2. Selected bond distances and angles for complex **d-1**

Bond distances (Å)		Bond angles (°)			
Dy1-O1	2.298(4)	O1-Dy1-O2	75.14(13)	O7-Dy2-O8	73.83(13)
Dy1-O2	2.366(4)	O1-Dy1-O4	78.89(13)	O7-Dy2-O10	128.09(13)
Dy1-O3	2.284(4)	O1-Dy1-O5	154.30(14)	O7-Dy2-O12	75.55(13)
Dy1-O4	2.343(4)	O1-Dy1-N6	131.23(14)	O7-Dy2-N3	73.58(13)
Dy1-O5	2.308(4)	O1-Dy1-N1	103.24(13)	O7-Dy2-N4	124.03(14)
Dy1-O6	2.376(4)	O1-Dy1-N2	71.75(14)	O8-Dy2-N3	81.90(14)
Dy1-N1	2.615(5)	O2-Dy1-O6	142.43(12)	O8-Dy2-N4	68.17(13)
Dy1-N2	2.616(5)	O2-Dy1-N1	69.49(14)	O9-Dy2-O7	85.91(13)
Dy2-O7	2.322(4)	O2-Dy1-N2	110.50(13)	O9-Dy2-O8	115.56(13)
Dy2-O8	2.353(4)	O3-Dy1-O1	82.76(13)	O9-Dy2-O10	75.61(13)
Dy2-O9	2.270(3)	O3-Dy1-O2	81.32(13)	O9-Dy2-O11	86.28(13)
Dy2-O10	2.339(4)	O3-Dy1-O4	75.91(14)	O9-Dy2-O12	77.42(13)
Dy2-O11	2.301(4)	O3-Dy1-O5	85.67(13)	O9-Dy2-N3	148.46(15)
Dy2-O12	2.345(4)	O3-Dy1-O6	122.87(13)	O9-Dy2-N4	147.57(14)
Dy2-N3	2.618(5)	O3-Dy1-N1	147.13(15)	O10-Dy2-O8	71.68(13)
Dy2-N4	2.613(4)	O3-Dy1-N2	147.23(14)	O10-Dy2-O12	141.91(12)
		O4-Dy1-O2	147.27(13)	O10-Dy2-N3	135.93(14)
		O4-Dy1-O6	70.30(13)	O10-Dy2-N4	75.57(13)
		O4-Dy1-N1	136.87(14)	O11-Dy2-O7	151.18(13)
		O4-Dy1-N2	79.18(13)	O11-Dy2-O8	134.07(13)
		O5-Dy1-O2	80.49(13)	O11-Dy2-O10	76.21(13)
		O5-Dy1-O4	120.26(13)	O11-Dy2-O12	75.68(13)
		O5-Dy1-O6	74.04(13)	O11-Dy2-N3	100.15(13)

	O5-Dy1-N1	75.15(13)	O11-Dy2-N4	72.66(13)
	O5-Dy1-N1	125.66(13)	O12-Dy2-O8	145.47(13)
	O6-Dy1-N1	77.48(14)	O12-Dy2-N3	74.45(14)
	O6-Dy1-N2	66.10(13)	O12-Dy2-N4	118.91(13)
	N1-Dy1-N2	61.63(14)	N3-Dy2-N4	61.88(14)

Table S3. Selected bond distances and angles for complex **l-1**

**Bond distances (Å)**		**Bond angles (º)**			
Dy1-O1	2.294(6)	O1-Dy1-O2	75.3(2)	O7-Dy2-O8	74.1(2)
Dy1-O2	2.378(6)	O1-Dy1-O4	78.8(2)	O7-Dy2-O10	128.2(2)
Dy1-O3	2.281(6)	O1-Dy1-O5	154.6(2)	O7-Dy2-O12	75.6(2)
Dy1-O4	2.354(6)	O1-Dy1-O6	131.2(2)	O7-Dy2-N3	73.8(2)
Dy1-O5	2.303(6)	O1-Dy1-N1	103.7(2)	O7-Dy2-N4	123.9(2)
Dy1-O6	2.376(6)	O1-Dy1-N2	71.6(2)	O8-Dy2-N3	82.2(2)
Dy1-N1	2.607(8)	O2-Dy1-N1	69.5(2)	O8-Dy2-N4	68.0(2)
Dy1-N2	2.617(8)	O2-Dy1-N2	110.5(2)	O9-Dy2-O7	85.6(2)
Dy2-O7	2.328(6)	O3-Dy1-O1	82.6(2)	O9-Dy2-O8	115.6(2)
Dy2-O8	2.354(6)	O3-Dy1-O2	81.4(2)	O9-Dy2-O10	75.6(2)
Dy2-O9	2.270(6)	O3-Dy1-O4	76.0(2)	O9-Dy2-O11	86.7(2)
Dy2-O10	2.336(6)	O3-Dy1-O5	85.8(2)	O9-Dy2-O12	77.3(2)
Dy2-O11	2.301(6)	O3-Dy1-O6	122.9(2)	O9-Dy2-N3	148.3(2)
Dy2-O12	2.344(6)	O3-Dy1-N1	147.1(2)	O9-Dy2-N4	148.0(2)
Dy2-N3	2.294(6)	O3-Dy1-N2	147.0(2)	O10-Dy2-O8	71.7(2)
Dy2-N4	2.378(6)	O4-Dy1-O2	147.5(2)	O10-Dy2-O12	141.7(2)
		O4-Dy1-O6	70.2(2)	O10-Dy2-N3	136.1(2)
		O4-Dy1-N1	136.8(2)	O10-Dy2-N4	75.9(2)
		O4-Dy1-N2	79.0(2)	O11-Dy2-O7	150.8(2)
		O5-Dy1-O2	80.6(2)	O11-Dy2-O8	134.1(2)
		O5-Dy1-O4	120.1(2)	O11-Dy2-O10	76.5(2)
		O5-Dy1-O6	73.9(2)	O11-Dy2-O12	75.3(2)
		O5-Dy1-N1	74.9(2)	O11-Dy2-N3	99.5(2)

	O5-Dy1-N2	125.8(2)	O11-Dy2-N4	72.8(2)
	O6-Dy1-O2	142.3(2)	O12-Dy2-O8	145.7(2)
	O6-Dy1-N1	77.3(2)	O12-Dy2-N3	74.3(2)
	O6-Dy1-N2	66.2(2)	O12-Dy2-N4	118.8(2)
	N1-Dy1-N2	62.0(2)	N4-Dy2-N3	61.6(2)

Table S4. Continuous Shape Measures[i] of the coordination sphere geometry for the Dy(III) centres in the complexes described in this work.

Compound		ETBPY	TT	JSD	BTPR	JBTPR	JETBPY	JGBF	TDD	SAPR	CU	HBPY	HPY	OP
**d-1**	**Dy1**	23.333	9.614	5.317	2.020	2.593	27.454	14.668	2.315	**0.686**	8.865	14.104	21.879	29.514
	**Dy2**	22.650	9.850	3.736	1.711	2.246	27.264	15.062	**1.085**	**1.083**	9.074	14.796	23.414	28.776
**l-1**	**Dy1**	23.466	9.581	5.304	2.059	2.583	27.623	14.749	2.357	**0.666**	8.860	14.187	21.953	29.512
	**Dy2**	22.659	9.784	3.744	1.697	2.269	27.313	15.041	**1.063**	**1.136**	9.006	14.632	23.212	28.982

ETBPY-8	13 D3h	Elongated trigonal bipyramid	TDD-8	6 D2d	Triangular dodecahedron
TT-8	12 Td	Triakis tetrahedron	SAPR-8	5 D4d	Square antiprism
JSD-8	11 D2d	Snub diphenoid J84	CU-8	4 Oh	Cube
BTPR-8	10 C2v	Biaugmented trigonal prism	HBPY-8	3 D6h	Hexagonal bipyramid
JBTPR-8	9 C2v	Biaugmented trigonal prism J50	HPY-8	2 C7v	Heptagonal pyramid
JETBPY-8	8 D3h	Johnson elongated triangular bipyramid	OP-8	1 D8h	Octagon
JGBF-8	7 D2d	Johnson gyrobifastigium J26			

Detailed *ab initio* calculations of complexes **2-4.**

The anisotropy axes of both Dy centres in **2**, **3** and **4** are collinear to each other with tilt angles of 28.80°, 0.002° and 0.045° respectively (Figure S1).For **2**, The eight KDs generated from the $^{6}H_{15/2}$spin-orbitstate span 505 and 540 cm^{-1} for Dy1 and Dy2 centres (Tables S5 and S6). The existence of two different Dy^{III} centres is confirmed from this different energy level splitting of the KDs. The ground state of both Dy centres is Ising type with the g_z value reaching the limit of the g_z value generated from pure |15/2> state. The axiality of

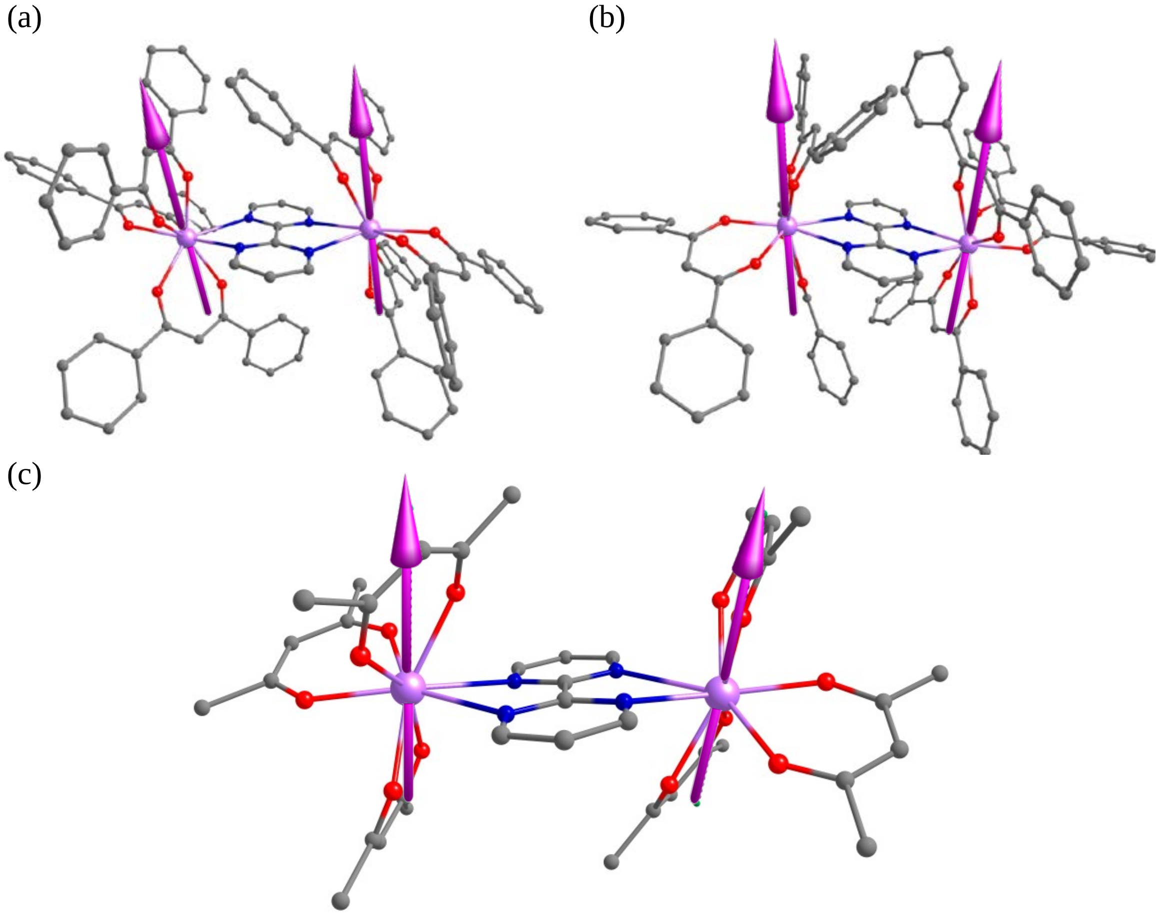

Figure S1: The anisotropy axis of both the Dy centres in **2** (a) **3** (b) and **4** (c). The methyl groups has been modelled by hydrogen in the three complexes. Hydrogens are omitted for clarity. Colour code: C-Grey, N-Blue, O-Red, Dy-Violet.

the KDs decreases as up to 5th KDs and then it starts to increase up to 8th KDs where it reaches its maximum, which is similar to the case of d-**1**. In the three cases, as observed for d-**1**, the g_{zz} axis is found to lie along the β-diketonate ligands at opposite sides of the Dy^{III} centre.

The relaxation mechanism of both Dy^{III} centres in **2**is depicted in Figure S2. Both Dy^{III} centres contain almost pure M_J = |±15/2> as ground state with negligible QTM. This is further corroborated by negative axial crystal field parameter (B_2^0).The matrix element between opposite pair of magnetic moments between ground and 1st excited KDs (Raman relaxation)for the Dy1 is very small (0.05 μ_B). This suggest possible Orbach relaxation via 1st excited KDs. Although the TA-QTM is considerably large (0.28 μ_B) at the first excited state and favours magnetic relaxation via this state, the matrix element of the next doublet is sufficiently large (2.73 μ_B) to reinforce the relaxation through KD3. This type of relaxation mechanism via higher excited doublets have been suggested earlier.[ii]

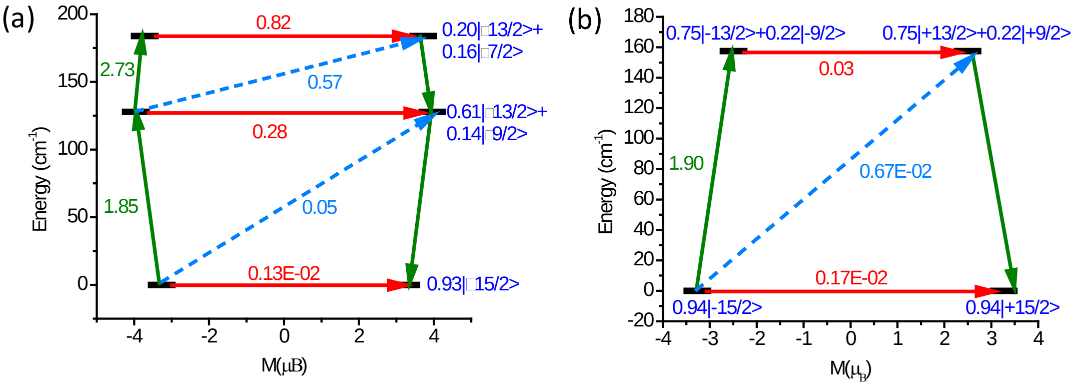

Figure S2: Relaxation mechanism of the Dy1 (a) and Dy2 (b) centres in **2.**The Black line indicates the KDs as function of magnetic moments. The red line represents QTM via ground states and TA-OTM via excited states. Dashed line indicates possible Orbach process.

The *ab initio* computed energy barrier 183.87 cm^{-1} is in accord with the experimental estimate. This is reflected by the fact that the non-axial crystal field parameter ($B_2^{2,}$ B_2^1, B_2^{-1}) are significantly larger than the axial crystal field parameter (B_2^0). Despite the above mentioned reasons, one must consider that the relaxation time obeys thermally activated relaxation pathways (Orbach process), which dominates only at high temperature, but at low temperature other relaxation process such as direct/Raman may dominate and creates the discrepancy between experimental and computed results. The computed QTM and TA-QTM for the Dy2 centre of complex **2** are considerably small, thus favouring the magnetic relaxation via KD3. However, the matrix element between 2nd and 3rd KDs with same magnetisation is very small that, forcing it to relax via the 1st excited state (the 7.25° angle between the anisotropy axis of the ground and first excited state is large enough as to force the relaxation to occurs through KD1), leading to an estimated barrier height of 157.48 cm^{-1}. The two different barrier heights computed for different Dy(III)

centres is consistent with experimental estimations, although the magnitudes are different. The computed non-axial crystal field parameter becomes larger than the axial crystal field parameters (Table S8), strengthening the probability of QTM in the ground state and decreasing the barrier height estimated by *ab initio* calculations. The Dy2 centre with larger distortion in D_{2d} (TDD-8) geometry is expected to have a lower barrier height for magnetisation reversal when compared to the Dy1 centre.

To decipher to role of exchange into magnetic anisotropy, the experimental magnetic susceptibility was fitted by Lines approach using POLY_ANISO programme (Figure S3). Although the magnetic anisotropy axes of both Dy^{III} centres are parallel to each other, the best fit is obtained using the J_{tot}= -0.047 cm^{-1} (J_{exch} = -0.04 cm^{-1} and $J_{dipolar}$ = -0.007 cm^{-1}). The deviation of the computed magnetic susceptibility with regard to the experimental one originates from the QTM that suppresses the single ion magnetisation blocking barrier of the Dy2 centre. The considerable tunnel splitting $5.1*10^{-6}$ blocks the magnetisation blockade in the third excited exchange coupled state (see Table S17 for the spectrum of lowest exchange levels). The extracted magnetisation blockade 127 cm^{-1} by Lines model resembles the energy of the 2nd KDs of the Dy1 centre (127 cm^{-1}).

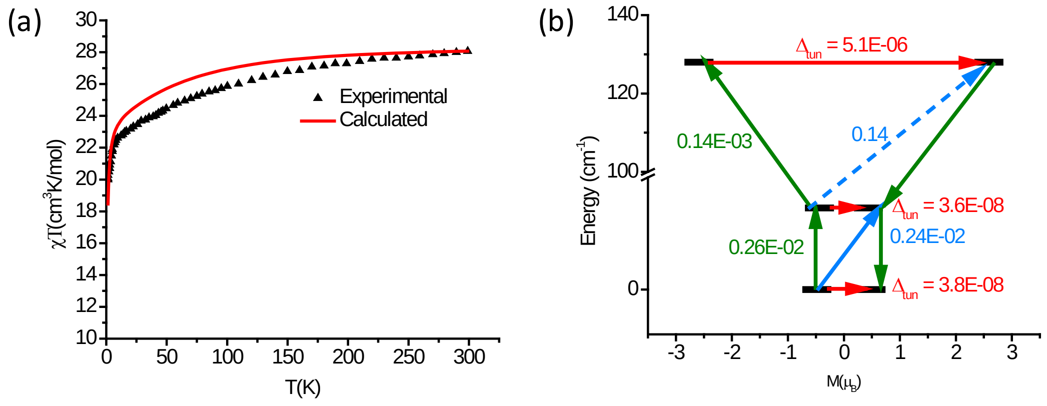

Figure S3: (a) Fitted experimental magnetic susceptibility using Lines model. (b) POLY_ANISO computed blockade barrier of **2**. The black lines indicate exchange states that have been arranged in compliance with the value of its magnetic moment. The red arrows (and pertinent values) correspond to tunnelling transitions within ground-state and first excited-state exchange doublets. However, olive and dashed arrows and their corresponding values represent transition magnetic moment matrix elements of spin-phonon relaxation pathways.

The complex **3** shows different magnetic relaxation compared to complex **2** due to the different lattice solvents (CH_3Cl for **2** and CH_3CN for **3**). Apart from this, the Dy^{III} centre in **3** has different distortion parameter from D_{2d} and D_{4d} symmetry compared to **2**. The anisotropy axes of both centres in **3**are shown in Figure 9. They are tilted by only 0.02°.

The computed g tensors along with energy spectrum and angle of the anisotropy axis of the excited state with ground state is gathered in Table S7-S8. The two Dy^{III}centres in this complex has same energy span generated from $^6H_{15/2}$ state, thus confirming that the two Dy^{III} centres have the same distortion parameter from D_{2d} and D_{4d} symmetry. The Ising ground state of the metal centre has g_z value close to that generated from the |15/2> state. The negative B_2^0 parameter obtained from crystal field analysis further corroborates the isolation of |15/2> as ground state (Table S13). The wave function analysis of the 1st and 2nd KDs is shown along with the relaxation mechanism (Figure S4). The ground state is consists of 0.91|15/2> state with a negligible QTM while the excited state has a major contribution from |13/2> state. The small TA-QTM (0.08μ_B) in the first excited KDs reinforces relaxation via upper excited states. The matrix element between 2ndand 3rd KDs is considerably large (2.86 μ_B), which favours the Orbach relaxation via 3rd KDs. The probability of Raman relaxation between 1st and 2nd KDs is very small (0.01μ_B) , as well as the angle between ground and 1st excited KDs is very small (around 6°), indicating that the relaxation via 1st excited state is ruled out.

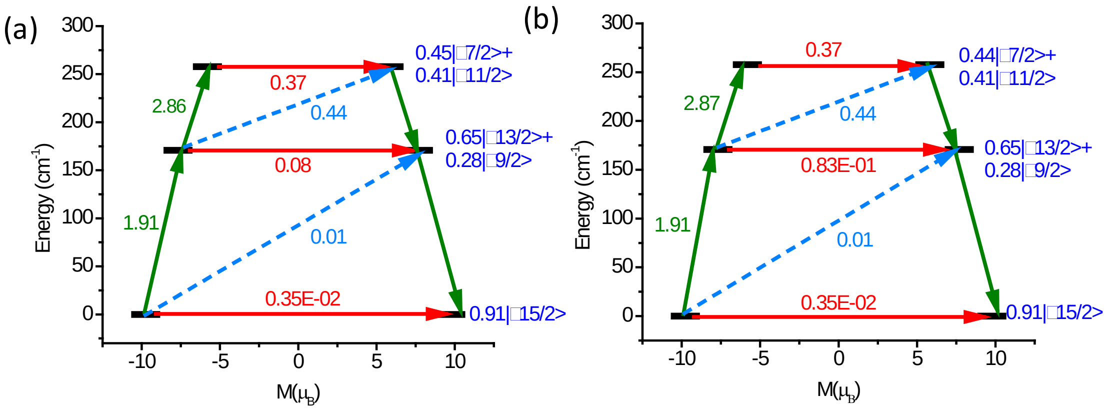

Figure S4: (a) Relaxation mechanism of the Dy1 centre in **3**. (b) Relaxation mechanism of the Dy2 centre in **2.**The Black line indicates the KDs as function of magnetic moments. The red line represents QTM via ground states and TA-OTM via excited states. Dashed line indicates possible Orbach process.

The 3rd KDs of **3**is composed of |11/2> and |7/2> states with contribution of 0.41 and 0.45 respectively. This large mixing between the wave functions generates the large TA-QTM (0.37 μ_B) between 3rd KDs and reinforces relaxation via this state. The angle of the anisotropy axis between 3rd KDs and 1st KDs of 7.21° is enough, as in the case of **2**, to favour magnetic relaxation via upper KDs. The effective energy barrier for the magnetisation reversal is the energy gap between KD1 and KD3 (257.83 cm^{-1}). This value

is very close to the barrier height of 267 cm^{-1} as observed experimentally. Since the QTM and TA-QTM becomes very low (the axial crystal filed parameters are larger or comparable to non-axial crystal filed parameters) for the Dy^{III} centres, the computed values for both the Dy centres are in line with experimental values.

As in the former case, the experimental magnetic susceptibility was fitted by Lines model using POLY_ANISO programme (Figure S5). The best fit is obtained using J_{tot} = 0.0059 cm^{-1} (J_{exch} = 0.006 cm^{-1} and $J_{dipolar}$ = -0.00001 cm^{-1}). The simulated susceptibility matches quite well with the experiment as the experimental barrier height is close to the *ab initio* computed barrier height that removes the possibility of QTM in the ground state. The tunnel splitting in the third exchange coupled excited state is considerably large ($1.8*10^{-6}$) to block the magnetisation via this state. The blocking barrier using Lines model (171.79 cm^{-1}) is far away from the experimental barrier height (267 cm^{-1}) (See Figure S5 and Table S18 for the spectrum of lowest exchange levels). Weaker coupling between the Dy^{III} ions suggest that the magnetic blockade observed arise essentially due to single-ion behaviour and coupled regime is important only extremely low temperatures.

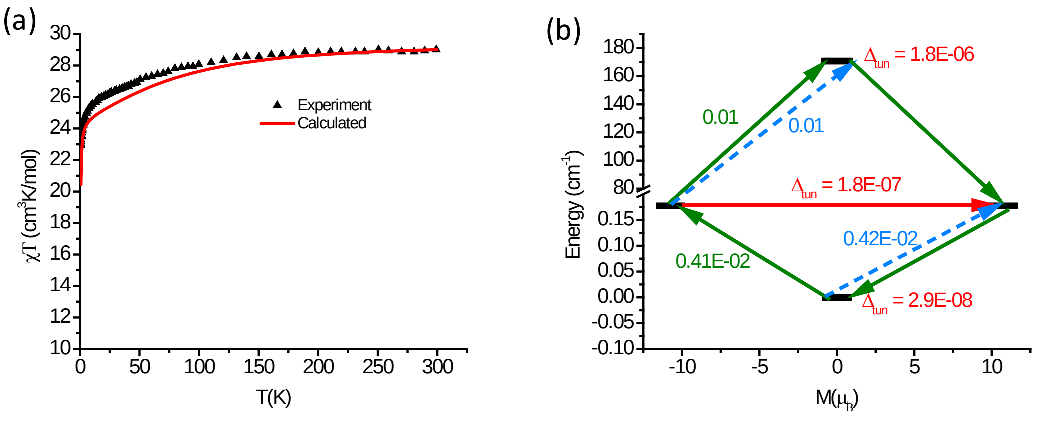

Figure S5: (a) Fitted experimental magnetic susceptibility using Lines model of complex **3**. (b) POLY_ANISO computed blockade barrier of**3**. The black lines indicate exchange states that have been arranged in compliance with the value of its magnetic moment. The red arrows (and pertinent values) correspond to tunnelling transitions within ground-state and first excited-state exchange doublets. However, olive and dashed arrows and their corresponding values represent transition magnetic moment matrix elements of spin-phonon relaxation pathways.

Finally, *ab initio* calculations on complex **4** show that the ground state is strongly anisotropic as depicted earlier. The computed energy spectrum along with the g tensors is gathered in Table S9-S10. The energy of the eight KDs span up to 534 cm^{-1} due to

moderate ligand field. The transverse anisotropic component in the ground KDs is negligible as reflected by the negligible QTM in the corresponding relaxation mechanism (Figure S6). The QTM is the lowest among all the molecules studied here. The axiality of the KDs starts to decrease up to 4th KDs and then starts to increase again to reach a maximum at 8th KDs.

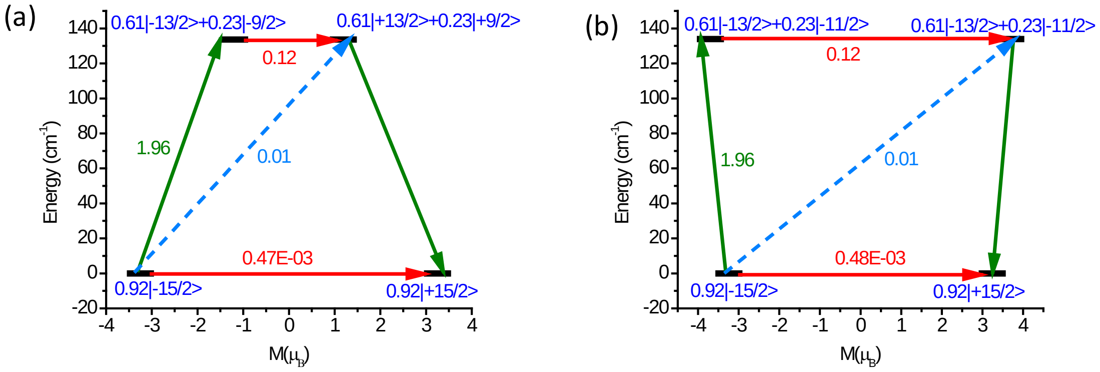

Figure S6: (a) Relaxation mechanism of the Dy1 centre in **4**. (b) Relaxation mechanism of the Dy2 centre in **2.**The Black line indicates the KDs as function of magnetic moments. The red line represents QTM via ground states and TA-OTM via excited states. Dashed line indicates possible Orbach process.

The transverse component of anisotropy in the 2nd KDs is very large and it is reflected by the TA-QTM of the 1st excited state, which is considerably large (0.12 μ_B) (Figure S6). The ground state KDs is composed of the |15/2> state and its isolation as ground state further confirmed from the negative B_2^0 parameter in the crystal field analysis (Table S 14). At the 1st excited KDs, the large mixing of the wave function (0.61 |13/2> + 0.23 |11/2>) generates considerable TA-QTM. The large angle (20.93°) between the anisotropy axis of ground and 1st excited KDs reinforces that relaxation takes place through KD2. Since the computed non-axial crystal field parameters become comparable to the axial crystal field parameters, the computed barrier height 133 cm^{-1} is overestimated with regard to the experimental barrier height of 97 cm^{-1} for magnetisation reversal. Due to the similar distortion parameters, the two Dy$^{II\ I}$centres follow the same relaxation pathways.

The magnetic exchange has been simulated by Lines approach using the POLY_ANISO as has been discussed earlier. The best fit is obtained using J_{tot} = 0.008 cm^{-1} (J_{exch} = 0.008 cm^{-1} and $J_{dipolar}$ = 0.001 cm^{-1}) (Figure S7). These values indicate, as expected, a very weak exchange between the Dy^{III} ions through the bipyrimidine bridging ligand. This

ferromagnetic exchange is in line with the anisotropy axis of the two Dy^{III} centres, which are parallel to each other with tilt angle of 0.045°.

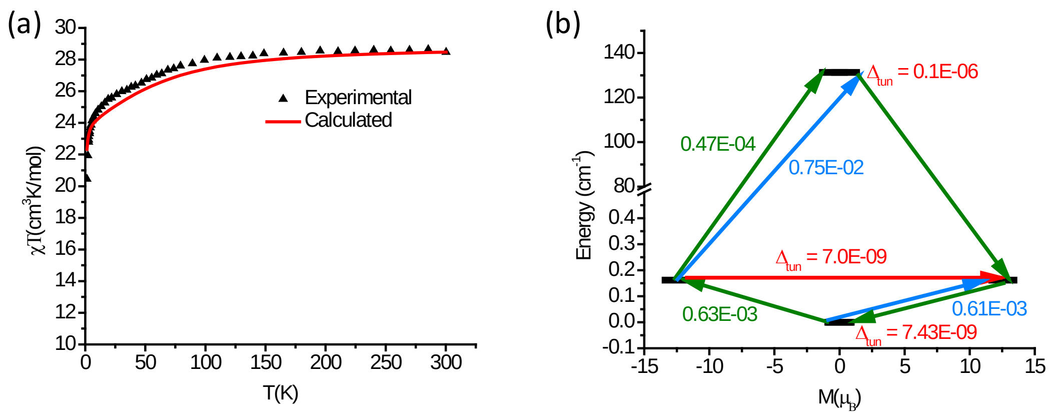

Figure S7: (a) Fitted experimental magnetic susceptibility using Lines model of complex **4**. (b) POLY_ANISO computed blockade barrier of **4**. The black lines indicate exchange states that have been arranged in compliance with the value of its magnetic moment. The red arrows (and pertinent values) correspond to tunnelling transitions within ground-state and first excited-state exchange doublets. However, olive and dashed arrows and their corresponding values represent transition magnetic moment matrix elements of spin-phonon relaxation pathways.

The significant tunnel splitting $0.1*10^{-6}$ in the third exchange coupled state favours the relaxation via this state (see Table S19 for the spectrum of lowest exchange levels). The simulated energy barrier of 131 cm^{-1} is close to that observed of 133 cm^{-1} from the single ion analysis, which is due to the weak coupling between two Dy^{III} ions.

Table S5. Computed energy spectrum along with g-tensors and the angle of the anisotropy axis of excited KDs with the ground KDs of Dy1 centre of complex **2**.

Energy (cm^{-1})	g_x	g_y	g_z	Angle of g_{zz} between ground and higher excited KDs(°)
0.00	0.001	0.007	19.609	--
127.88	0.544	0.832	16.113	26.93
183.87	1.519	2.701	14.006	51.61
232.50	1.008	3.826	11.895	44.82
282.28	4.221	4.852	11.887	84.40
383.24	0.404	1.454	15.923	63.12
428.74	0.533	2.109	16.187	74.67
505.14	0.102	0.208	19.266	58.03

Table S6. Computed energy spectrum along with g tensors and the angle of the anisotropy axis of excited KDs with the ground KDs of Dy2 centre of complex **2**.

Energy (cm^{-1})	g_x	g_y	g_z	Angle of g_{zz} between ground and higher excited KDs(°)
0.00	0.004	0.006	19.588	--
157.48	0.056	0.096	15.993	7.25
229.08	0.773	0.987	13.555	32.55
275.97	1.756	3.481	9.732	36.36
307.83	3.356	5.683	12.273	79.76
388.83	0.193	0.275	19.328	65.62
428.51	0.037	0.065	17.762	78.15
540.73	0.018	0.044	19.281	55.14

Table S7. Computed energy spectrum along with g tensors and the angle of the anisotropy axis of excited KDs with the ground KDs of Dy1 centre of complex **3**.

Energy (cm^{-1})	g_x	g_y	g_z	Angle of g_{zz} between ground and higher excited KDs(°)
0.00	0.008	0.013	19.483	--
170.80	0.152	0.302	15.437	6.02
257.83	0.924	1.155	11.695	7.28
310.55	1.274	2.878	8.818	36.46
331.43	11.328	7.196	2.893	8.26
440.83	0.080	0.143	19.512	58.72
496.04	0.017	0.098	17.541	95.02
549.49	0.039	0.112	18.503	121.43

Table S8. Computed energy spectrum along with g tensors and the angle of the anisotropy axis of excited KDs with the ground KDs of Dy2 centre of complex **3**.

Energy (cm^{-1})	g_x	g_y	g_z	Angle of g_{zz} between ground and higher excited KDs(°)
0.00	0.008	0.013	19.525	--
170.80	0.152	0.302	15.471	6.12
257.83	0.924	1.151	11.712	7.21
310.55	1.275	2.883	8.826	36.33
331.43	11.327	7.210	2.895	8.14
440.83	0.080	0.142	19.494	58.76
496.05	0.018	0.098	17.555	95.16
549.49	0.039	0.112	18.524	121.62

Table S9. Computed energy spectrum along with g tensors and the angle of the anisotropy axis of excited KDs with the ground KDs of Dy1 centre of complex **4**.

Energy (cm^{-1})	g_x	g_y	g_z	Angle of g_{zz} between ground and higher excited KDs(°)
0.00	0.000	0.002	19.493	--
133.70	0.274	0.331	15.658	20.96
187.29	1.337	1.627	13.938	151.26
222.66	3.120	4.749	9.300	148.33
272.72	2.848	2.639	9.741	61.30
317.91	0.852	1.632	16.983	109.99
446.03	0.027	0.027	17.630	80.50
534.30	0.007	0.019	18.973	55.60

Table S10. Computed energy spectrum along with g tensors and the angle of the anisotropy axis of excited KDs with the ground KDs of Dy2 centre of complex **4**.

Energy (cm^{-1})	g_x	g_y	g_z	Angle of g_{zz} between ground and higher excited KDs(°)
0.00	0.001	0.002	19.446	--
133.93	0.277	0.331	15.616	20.97
187.85	1.304	1.590	13.862	28.30
223.10	3.181	4.796	9.319	147.69
273.30	2.720	2.601	9.726	61.47
317.66	0.881	1.716	16.912	110.16
446.51	0.026	0.027	17.597	80.51
534.60	0.007	0.020	18.942	55.78

Table S11. SINGLE_ANISO computed crystal field parameters for the Dy1 and Dy2 centres in complex d-**1**. The crystal field Hamiltonian:

$$\hat{H}_{CF} = \sum \sum_{k=-q}^{q} B_k^q \tilde{O}_k^q$$

Where $\tilde{O}_k^q$ and$B_k^q$are the computed extended Stevens operator and crystal field (CF) parameter, respectively. Quantization axis is considered as the main magnetic axis of the KD1

k	q	B_k^q (Dy1)	B_k^q (Dy2)
2	-2	-6.89E-01	2.84E-02
	-1	-3.02E+00	1.89E-01
	0	**-1.66E+00**	**-1.46E+00**
	1	2.17E+00	3.62E+00
	2	-2.54E-01	**1.70E+00**
4	-4	-2.03E-02	1.35E-02
	-3	-3.70E-02	3.12E-02
	-2	-2.11E-02	7.81E-03
	-1	2.39E-02	1.20E-02

	0	**-5.31E-03**	**-5.26E-03**
	1	3.04E-03	-1.33E-02
	2	7.88E-03	2.31E-02
	3	-5.78E-03	4.50E-03
	4	2.05E-03	**-1.34E-02**
6	-6	1.04E-04	1.22E-04
	-5	-4.40E-04	5.42E-04
	-4	-1.01E-04	1.23E-04
	-3	-3.57E-04	2.40E-04
	-2	1.02E-04	-6.99E-05
	-1	-1.50E-04	-1.73E-04
	0	**7.05E-06**	**1.96E-06**
	1	-1.91E-04	-3.85E-05
	2	-7.41E-05	-6.74E-05
	3	-1.41E-04	2.47E-04
	4	-9.58E-05	7.10E-05
	5	-1.93E-04	-5.88E-04
	6	-6.94E-05	**3.07E-05**

Table S12. SINGLE_ANISO computed crystal field parameters for the Dy1 and Dy2 centres in complex **2**. The crystal field Hamiltonian:

$$\hat{H}_{CF} = \sum \sum_{k=-q}^{q} B_k^q \tilde{O}_k^q$$

Where $\tilde{O}_k^q$ and$B_k^q$are the computed extended Stevens operator and crystal field (CF) parameter, respectively. Quantization axis is considered as the main magnetic axis of the KD1

k	q	B_k^q (Dy1)	B_k^q (Dy2)
2	-2	8.98E-01	-1.34E+00
	-1	2.46E+00	-7.65E-01
	0	**-1.65E+00**	**-1.78E+00**
	1	2.10E+00	2.70E+00
	2	1.14E+00	**7.66E-01**
4	-4	-9.57E-03	9.29E-03
	-3	2.70E-02	-2.63E-02
	-2	-1.09E-02	-3.03E-02
	-1	-2.14E-02	-9.59E-03
	0	**-4.60E-03**	**-4.56E-03**
	1	4.83E-03	-9.29E-03
	2	2.03E-02	1.98E-03
	3	7.37E-02	5.51E-03
	4	4.85E-04	**-5.87E-03**
6	-6	5.57E-06	1.10E-04
	-5	-3.93E-04	3.52E-04
	-4	2.73E-05	-1.09E-04
	-3	-1.61E-04	-1.71E-04
	-2	1.43E-04	6.41E-05
	-1	3.32E-05	1.37E-04
	0	**1.71E-06**	**-8.45E-06**
	1	-9.68E-05	-2.00E-05
	2	3.01E-05	9.38E-05
	3	2.18E-04	-2.64E-04
	4	1.22E-04	-1.45E-04
	5	3.45E-04	-4.01E-06
	6	2.30E-04	**-1.76E-05**

Table S13. SINGLE_ANISO computed crystal field parameters for the Dy1 and Dy2 centres in complex **3**. The crystal field Hamiltonian:

$$\hat{H}_{CF} = \sum \sum_{k=-q}^{q} B_k^q \tilde{O}_k^q$$

Where $\tilde{O}_k^q$ and$B_k^q$are the computed extended Stevens operator and crystal field (CF) parameter, respectively. Quantization axis is considered as the main magnetic axis of the KD1

k	q	B_k^q (Dy1)	B_k^q (Dy2)
2	-2	3.61E-01	3.70E-01
	-1	3.49E-01	3.62E-01
	0	**-1.92E+00**	**-1.92E+00**
	1	1.11E+00	1.15E+00
	2	**1.75E+00**	**1.75E+00**
4	-4	1.05E-03	7.63E-04
	-3	3.40E-02	3.34E-02
	-2	2.90E-02	2.92E-02
	-1	5.53E-03	5.83E-03
	0	**-4.46E-03**	**-4.47E-03**
	1	7.69E-05	5.42E-04
	2	2.32E-02	2.30E-02
	3	1.27E-02	1.18E-02
	4	**-1.66E-02**	**-1.67E-02**
6	-6	8.32E-05	8.15E-05
	-5	2.80E-04	2.78E-04
	-4	1.97E-04	1.96E-04
	-3	1.06E-04	1.11E-04
	-2	-1.64E-04	-1.62E-04
	-1	-5.15E-05	-5.36E-05
	0	**-1.80E-05**	**-1.81E-05**
	1	-7.75E-05	-7.44E-05
	2	8.55E-05	8.51E-05
	3	-1.26E-04	-1.29E-04
	4	-5.31E-06	-8.38E-06
	5	2.39E-04	2.34E-04
	6	**-1.09E-04**	**-1.10E-04**

Table S14. SINGLE_ANISO computed crystal field parameters for the Dy1 and Dy2 centres in complex **4**. The crystal field Hamiltonian:

$$\hat{H}_{CF} = \sum \sum_{k=-q}^{q} B_k^q \tilde{O}_k^q$$

Where $\tilde{O}_k^q$ and$B_k^q$are the computed extended Stevens operator and crystal field (CF) parameter, respectively. Quantization axis is considered as the main magnetic axis of the KD1

k	q	B_k^q (Dy1)	B_k^q (Dy2)
2	-2	4.76E-01	6.10E-01
	-1	-3.50E+00	3.40E+00
	0	**-1.65E+00**	**-1.65E+00**
	1	-1.23E+00	1.42E+00
	2	-1.11E+00	**-1.06E+00**
4	-4	1.18E-02	1.34E-02
	-3	-1.66E-02	2.74E-02

	-2	3.20E-02	3.18E-02
	-1	1.05E-02	-1.13E-02
	0	**-3.17E-03**	**-3.18E-03**
	1	-1.32E-02	1.24E-02
	2	-1.24E-03	2.51E-03
	3	6.11E-02	-5.74E-02
	4	-8.29E-03	**-5.51E-03**
6	-6	-2.88E-05	-4.55E-05
	-5	7.18E-05	-9.87E-05
	-4	-8.05E-05	-4.22E-05
	-3	-2.92E-04	2.99E-04
	-2	-1.00E-04	-8.62E-05
	-1	8.35E-05	-7.12E-05
	0	**-1.79E-05**	**-1.76E-05**
	1	1.79E-04	-1.82E-04
	2	-1.21E-04	-1.34E-04
	3	8.15E-05	-3.20E-05
	4	-1.58E-04	-1.73E-04
	5	-9.12E-05	6.76E-05
	6	5.66E-05	**4.31E-05**

Table S15. Exchange values of complexes d-**1** to **4** using DFT approach.

Complex	J (cm^{-1})
d-**1**	-0.0008
**2**	-0.0008
**3**	-0.0008
**4**	-0.0011

Table S16. Energies (cm^{-1}), corresponding tunnel splitting (Δ_{tun}) and g values (main magnetic g factors) of the low-lying exchange doublets state in complex d-**1**. We have only summarised the exchange doublets through which eventual magnetization relaxation takes place:

Ising doublets	Energy (cm^{-1})	g values			Δ_{tun} (cm^{-1})
		g_{xx}	g_{yy}	g_{zz}	
1	0.0000<br>0.0000	3.2*10^{-8}	1.2*10^{-8}	1.5517	2.4*10^{-7}
2	0.2073<br>0.2073	3.2*10^{-8}	4.7*10^{-8}	39.3413	9.3*10^{-7}
3	129.9713<br>129.9713	3.2*10^{-8}	4.4*10^{-8}	4.4843	0.6*10^{-5}

Table S17. Energies (cm^{-1}), corresponding tunnel splitting (Δ_{tun}) and g values (main magnetic g factors) of the low-lying exchange doublets state in complex **2**. We have only

summarised the exchange doublets through which eventual magnetization relaxation takes place:

Ising doublets	Energy (cm^{-1})	g values			Δ_{tun} (cm^{-1})
		g_{xx}	g_{yy}	g_{zz}	
1	0.0000<br>0.0000	$3.2*10^{-8}$	0.0	0.9698	$3.8*10^{-8}$
2	0.3636<br>0.3636	$3.2*10^{-8}$	$0.8*10^{-8}$	39.1838	$3.6*10^{-8}$
3	128.0021<br>128.0021	$1.7*10^{-8}$	$1.0*10^{-7}$	9.3951	$5.1*10^{-6}$

Table S18. Energies (cm^{-1}), corresponding tunnel splitting (Δ_{tun}) and g values (main magnetic g factors) of the low-lying exchange doublets state in complex **3**. We have only summarised the exchange doublets through which eventual magnetization relaxation takes place:

Ising doublets	Energy (cm^{-1})	g values			Δ_{tun} (cm^{-1})
		g_{xx}	g_{yy}	g_{zz}	
1	0.0000<br>0.0000	0.0	$0.6*10^{-8}$	0.0756	$2.9*10^{-8}$
2	0.1772<br>0.1772	$3.4*10^{-7}$	$6.7*10^{-7}$	39.0082	$1.8*10^{-7}$
3	170.7947<br>170.4947	$0.2*10^{-8}$	$0.4*10^{-8}$	0.3125	$1.8*10^{-6}$

Table S19. Energies (cm-1), corresponding tunnel splitting (Δ_{tun}) and g values (main magnetic g factors) of the low-lying exchange doublets state in complex **4**. We have only summarised the exchange doublets through which eventual magnetization relaxation takes place:

Ising doublets	Energy (cm^{-1})	g values			Δ_{tun} (cm^{-1})
		g_{xx}	g_{yy}	g_{zz}	
1	0.0000<br>0.0000	0.1*10-8	0.2*10-8	0.0891	$7.4*10^{-9}$
2	0.1617<br>0.1617	3.2*10-8	6.1*10-8	38.8336	$7.0*10^{-9}$
3	131.2893<br>131.2893	3.2*10-8	0.4*10-8	7.4691	$0.1*10^{-6}$

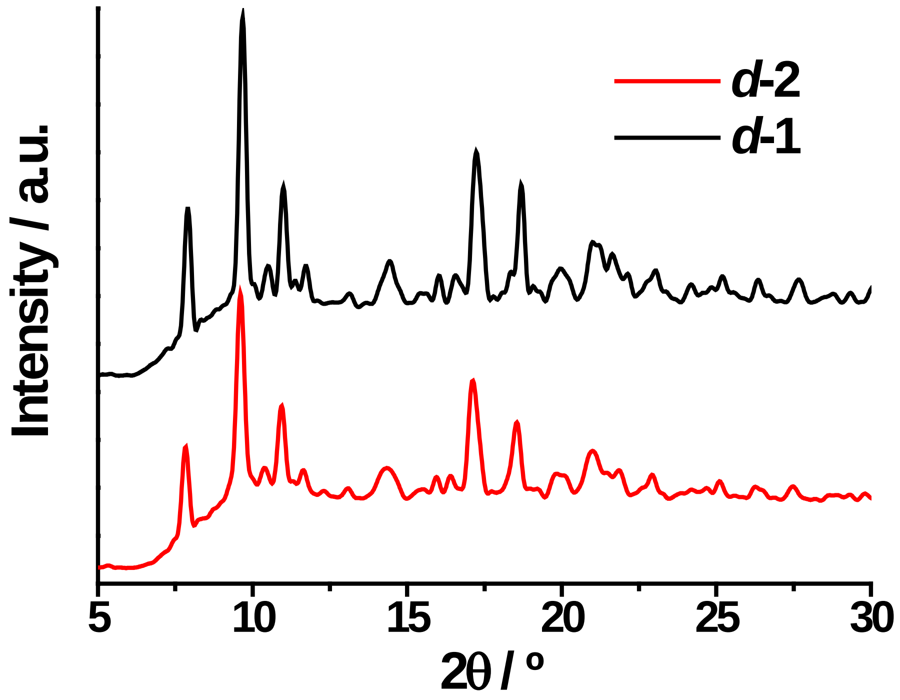

Figure S8. Experimental XRPD diagrams for complexes **d-1** (black) and **d-2** (red).

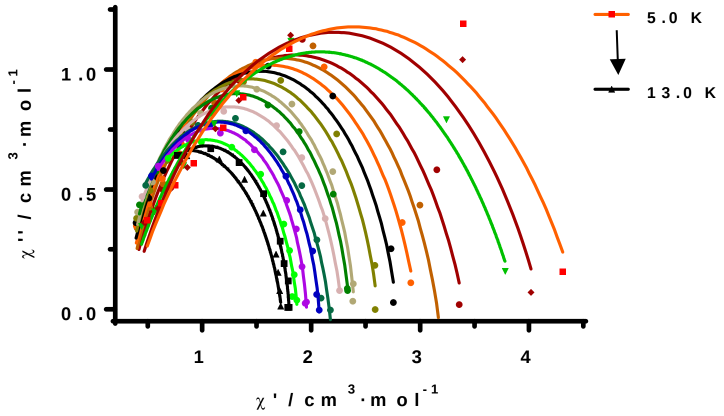

Figure S9. Cole-Cole plot for**d-1** at zero field.

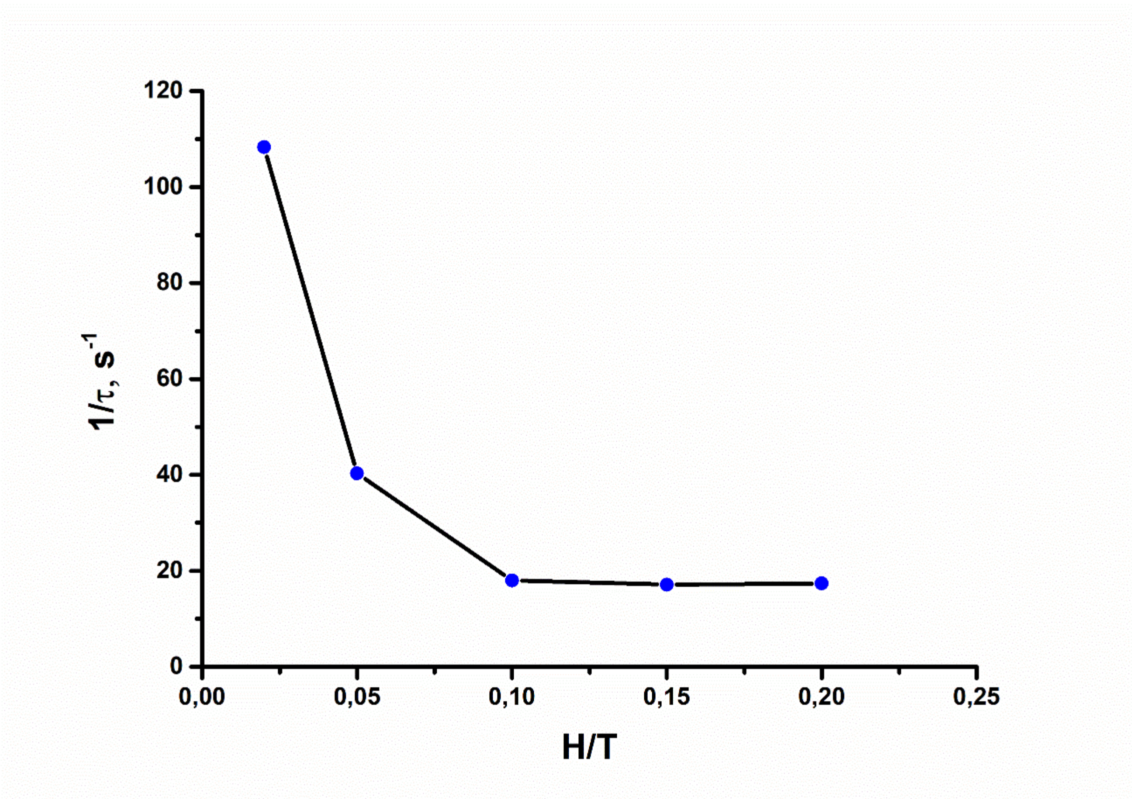

Figure S10. Field dependence of the relaxation time for **d-1** at 5 K.

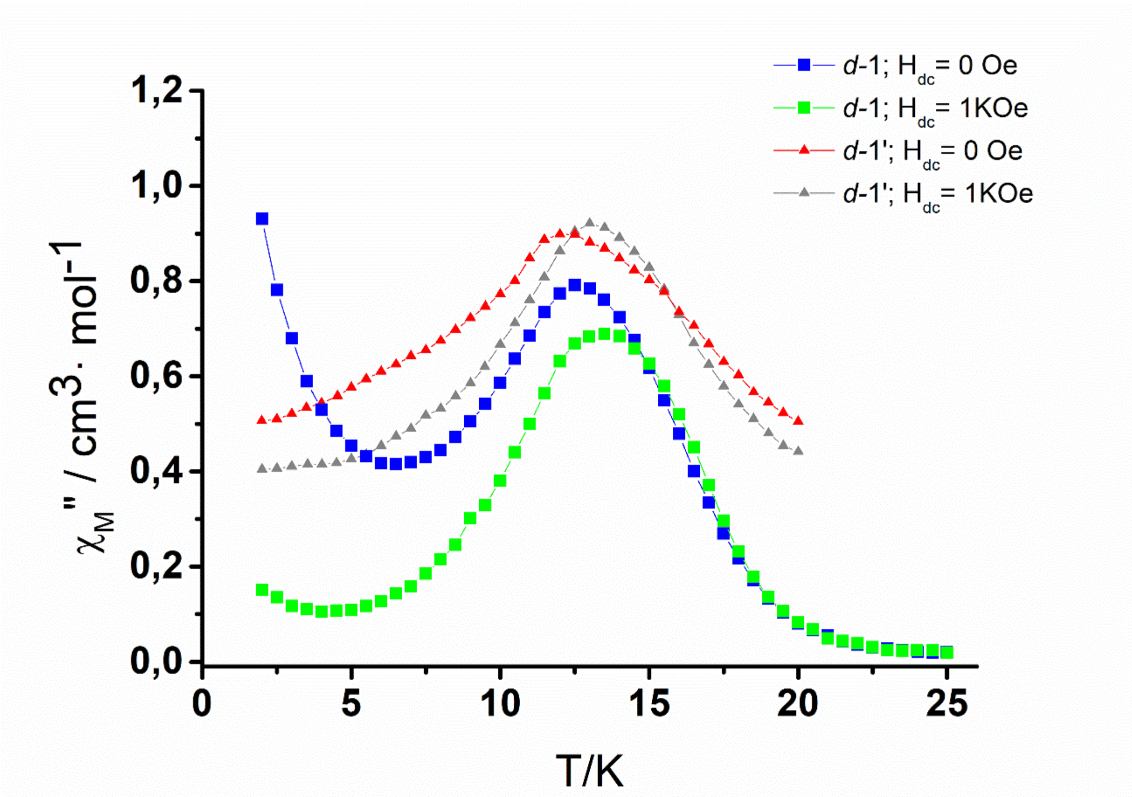

Figure S11. Thermal dependence of the out-phase susceptibilities under zero and 0.1 T field for complex d-**1** and the diluted complex d-**1’**at 1400 Hz.

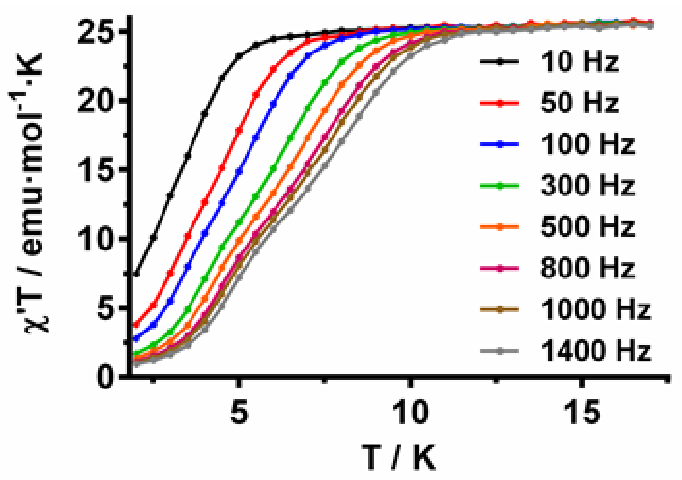

Figure S12. Thermal dependence of the $\chi'_M T$ product for complex **1** under an optimized dc field of 0.1 T.

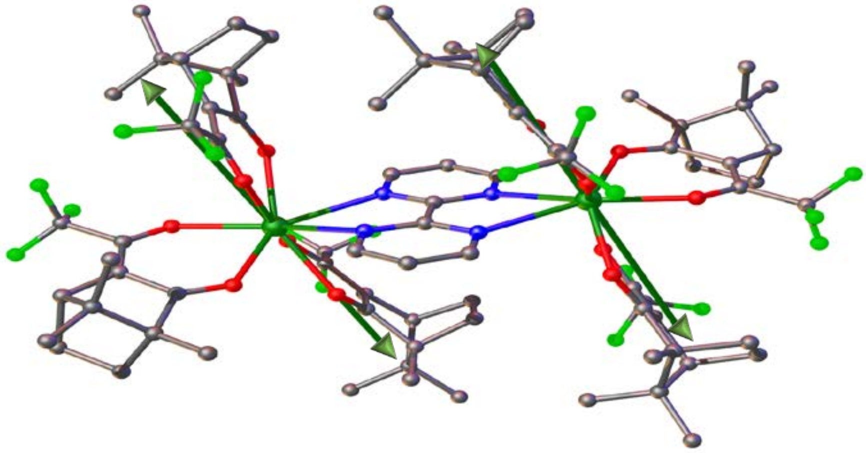

Figure **S13**. Quantitative calculation of the anisotropic axes orientation (green arrows) using the Chilton´s method.[ii]

---

[i] a) S. Alvarez, P. Alemany, D. Casanova, J. Cirera, M. Llunell, D. Avnir, *Coord. Chem. Rev.* **2005**, *249*, 1693-1708, b) J. Cirera, E. Ruiz, S. Alvarez, *Organometallics*, **2005**, *24*, 1556-1562.

[ii] Singh, S. K., Gupta, T., Shanmugam, M., Rajaraman, G. (2014). Unprecedented magnetic relaxation via the fourth excited state in low-coordinate lanthanide single-ion magnets: a theoretical perspective. Chem. Commun. 50, 15513-15516.

[iii] N. F. Chilton, R. P. Anderson, L. D. Turner, A. Soncini and K. S. Murray, J. Comput. Chem., 2013, 34, 1164−1175.
